# Ocean Alkalinity, Buffering and Biogeochemical Processes

**DOI:** 10.1029/2019RG000681

**Published:** 2020-09

**Authors:** Jack J. Middelburg, Karline Soetaert, Mathilde Hagens

**Affiliations:** ^1^ Department of Earth Sciences, Geosciences Utrecht University Utrecht The Netherlands; ^2^ Department of Estuarine and Delta Systems Royal Netherlands Institute for Sea Research (NIOZ Yerseke) and Utrecht University Yerseke The Netherlands; ^3^ Soil Chemistry and Chemical Soil Quality Wageningen University Wageningen The Netherlands

**Keywords:** alkalinity, carbon, buffering, biogeochemistry, modeling

## Abstract

Alkalinity, the excess of proton acceptors over donors, plays a major role in ocean chemistry, in buffering and in calcium carbonate precipitation and dissolution. Understanding alkalinity dynamics is pivotal to quantify ocean carbon dioxide uptake during times of global change. Here we review ocean alkalinity and its role in ocean buffering as well as the biogeochemical processes governing alkalinity and pH in the ocean. We show that it is important to distinguish between measurable titration alkalinity and charge balance alkalinity that is used to quantify calcification and carbonate dissolution and needed to understand the impact of biogeochemical processes on components of the carbon dioxide system. A general treatment of ocean buffering and quantification via sensitivity factors is presented and used to link existing buffer and sensitivity factors. The impact of individual biogeochemical processes on ocean alkalinity and pH is discussed and quantified using these sensitivity factors. Processes governing ocean alkalinity on longer time scales such as carbonate compensation, (reversed) silicate weathering, and anaerobic mineralization are discussed and used to derive a close‐to‐balance ocean alkalinity budget for the modern ocean.

## Key Points

- Titration and charge balance alkalinity differ
- The impact of biogeochemical processes on pH depends on environmental conditions
- Ocean alkalinity budget is balanced when the additional alkalinity input from riverine particulate inorganic carbon and sedimentary sources is included

## 1 Introduction

The ocean plays a major role in controlling atmospheric carbon dioxide and storage of anthropogenic carbon (Gruber et al., 2019). For the last decade, ocean uptake of anthropogenic carbon was 2.5 ± 0.6 Pg C year^−1^, that is, about 23% of annual anthropogenic carbon emissions due to fossil fuels, cement production, and land‐use change (Friedlingstein et al., 2019). The cumulative (1850–2019) total release of anthropogenic carbon was 655 ± 65 Pg C, of which 160 ± 20 Pg C (about 24%) has accumulated in the ocean (Friedlingstein et al., 2019). This crucial role of the ocean in attenuating the increase in atmospheric carbon dioxide, and thus global warming, is related to the large volume (and surface area) of the ocean and the reaction of dissolved carbon dioxide with water to form carbonic acid, a weak acid that dissociates to protons and the conjugated bases bicarbonate and carbonate, which are not directly exchangeable with the atmosphere (Butler, 1982). The redistributions among gaseous and dissolved carbon dioxide, carbonic acid, bicarbonate, and carbonate ions are governed by multiple co‐occurring equilibria with the result that approximately 19 out of the 20 molecules of carbon dioxide entering the ocean are converted into bicarbonate and carbonate ions. The total amount of dissolved inorganic carbon (DIC) in the ocean is typically about 200 times that of dissolved carbon dioxide (Middelburg, 2019; Zeebe & Wolf‐Gladrow, 2001).

This reequilibration following the principles of le Chatelier (1884) provides resistance to, but does not entirely eliminate, changes in ocean carbon chemistry. Oceanic uptake of anthropogenic carbon dioxide has caused increases in dissolved carbon dioxide and total inorganic carbon concentrations and decreases in carbonate ions and ocean pH, that is, ocean acidification (Gattuso & Hansson, 2011). Ocean acidification has consequences for further ocean carbon dioxide uptake, the precipitation and dissolution of carbonate minerals, and for the functioning and survival of marine organisms (Kroeker et al., 2013). It is therefore important that we understand and are able to quantify the buffering, that is, resistance, of the ocean in the changing world of the Anthropocene. Detailed understanding and quantification of how biogeochemical processes impact pH and marine carbon dioxide equilibria is pivotal to predicting the impact of ocean acidification on marine organisms, carbonate mineral precipitation and dissolution, (seasonal) variability in carbonate system parameters and the resilience of various ecosystem functions (Orr et al., 2018). Understanding is also required to use pH observations to infer the intensity and changes in biogeochemical processes and to evaluate the feasibility of ocean engineering options (Gattuso et al., 2018; Renforth & Henderson, 2017; Soetaert et al., 2007).

Although acid–base equilibria of simple solutions are well understood (Butler, 1964, 1982; Morel & Hering, 1993; Stumm & Morgan, 1981), the carbon dioxide system in seawater remains challenging because of the complexity of multiple equilibria (Zeebe & Wolf‐Gladrow, 2001). Alkalinity, the excess of bases (proton acceptors) over acids (proton donors) in a solution (a complete definition is provided in section 2), is not only impacted by acid–base additions but also by redox reactions and mineral dissolution and precipitation. Oxidation reactions involving oxygen generally consume alkalinity, while anaerobic processes usually produce alkalinity. Dissolution of minerals is often accompanied by alkalinity generation. Alkalinity is a central concept in our treatment of the oceanic carbon dioxide system, because it is measurable, it remains unchanged with pressure and temperature (i.e., it is conservative), and it is governed by the net effect of multiple chemical equilibria and often needed to solve the mathematical equilibrium problem (Butler, 1982; Stumm & Morgan, 1981). However, there are multiple interpretations, and even definitions, of alkalinity that are not always used in a consistent way. One of the goals of this review is to clarify inconsistencies or sources of confusion, for example, the distinction between titration alkalinity (that can be measured) and charge balance alkalinity (that should be used to interpret biogeochemical processes in nature). Another goal is to discuss approaches to quantify the resistance (buffering) or its inverse, i.e. the sensitivity of the ocean carbon dioxide system and pH to change. While many geochemical and oceanographic studies mention ocean buffering, there are few where buffer and/or sensitivity factors are being used, except for the well‐known Revelle factor expressing the sensitivity of pCO_2_ to changes in DIC (Bolin & Eriksson, 1959; Revelle & Suess, 1957; Sarmiento & Gruber, 2006; Sundquist et al., 1979). This is surprising as rigorous treatments of buffering have been published a century ago (Koppel & Spiro, 1914; van Slyke, 1922).

Following a treatment of ocean alkalinity (section 2) and sensitivity and buffer factors (section 3), we will discuss the impact of biogeochemical processes on pH and pCO_2_ (section 4), heterogeneous buffering, including carbonate compensation (section 5), and factors governing ocean alkalinity, including an alkalinity budget of the ocean (section 6). Basic terminology is explained in the Glossary. The supporting information accompanying this article contains three sections, and the R script used to generate figures and results.

## 2 Ocean Alkalinity

There is a long history from the eighteenth century observation that seawater is alkaline (Marsigli, 1725) to the modern concept of seawater alkalinity (Dickson, 1981; Zeebe & Wolf‐Gladrow, 2001). The term alkalinity was already in use by chemists in the first half of the nineteenth century (e.g., Donovan, 1839) and utilized since in multiple disciplines, including medicine (Andral, 1850) and oceanography (Dittmar, 1884). Dickson (1992) provides an excellent historical account on the alkalinity concept in seawater and showed that it involved both advances in analytical procedures as well as the development of a chemical model for seawater. Rather than recapitulating the historical context, we believe it is instructive to formally distinguish between titration alkalinity, that is, total alkalinity, as defined by Dickson (1981) and the charge‐balance alkalinity needed to quantify buffering and pH changes in natural environments. Observational and experimental studies in the ocean are normally based on titration alkalinity, but theoretical, modeling, and geological studies sometimes employ the charge balance approach (e.g., Broecker, 1974; Boudreau, 1996; Turchyn & DePaolo, 2019). The charge‐balance alkalinity concept is often used in freshwater systems (with high concentrations of dissolved organic matter) and is also known as the excess negative charge (ENC; Soetaert et al., 2007) and linked to the explicit conservative expression of total alkalinity (Wolf‐Gladrow et al., 2007; Zeebe & Wolf‐Gladrow, 2001). This difference between titration alkalinity (TA) and charge‐balance alkalinity (CBA) is related to the equations used to solve the chemical equilibrium problem: The TA is based on a proton balance, while CBA is based on a charge balance closure (supporting information S1). Depending on the specific problem at hand and definition of the system, TA and CBA may differ or be identical. The lack of distinction between TA and CBA has caused confusion and discussion.

### 2.1 Titration Alkalinity

In 1981, Dickson defined the alkalinity (TA) as follows: “*The total alkalinity of a natural water is thus defined as the number of moles of hydrogen ion equivalent to the excess of proton acceptors (bases formed from weak acids with a dissociation constant K ≤ 10* ^*‐4.5*^ *and zero ionic strength) over proton donors (acids with K > 10* ^*‐4.5*^ *) in one kilogram of sample*.” The definition is stated in gravimetric units to remain independent of the temperature and pressure of the system. Furthermore, Dickson (1981) adopted a pK value of 4.5 as the reference level to distinguish between proton donors (acids with a dissociation constant pK < 4.5) and proton acceptors (pK ≥ 4.5) to continue the common practice to match the reference level with the carbonic acid equivalence point of a titration.

**Figure 1:**
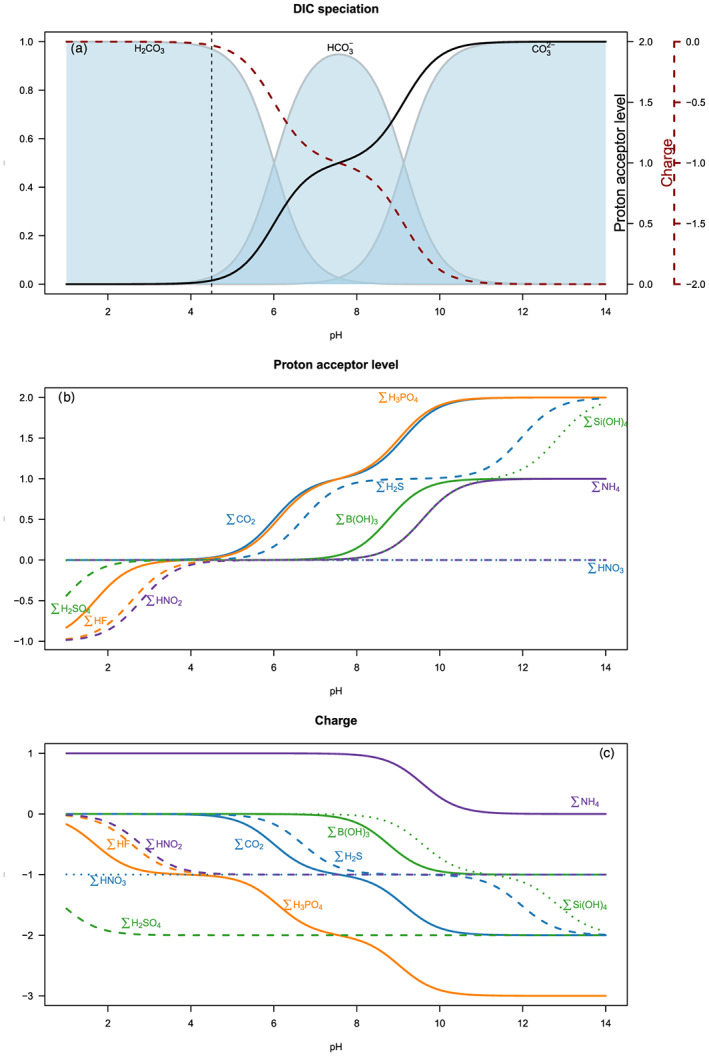
Speciation, proton acceptor levels, and charge as a function of pH. (a) Bjerrum plot showing the distribution of carbonic acid, bicarbonate, and carbonate as a function of pH and the corresponding proton acceptor level (solid black line) and charge (red dashed line); (b) the proton acceptor level for selected acid–base pairs; (c) the charge for selected acid–base pairs. Details of the calculations are presented in the supporting information.

Using this exact definition of alkalinity of Dickson (1981), it is straightforward to calculate the titration alkalinity for any system for which the contributing components are known and characterized in terms of dissociation constants. Figure 1a shows the distribution diagram of acid–base pairs (Bjerrum plot) for the carbonate system in seawater. For the CO_2_‐H_2_O system, at pH = 4.5, carbonic acid is by far the dominant species and used as reference. Referenced to this point, we then arrive at the proton balance, a mass balance for protons (see supporting information S1)

(1) $$H^{+}={{\mathrm{HCO}}_{3}}^{-}+{{\text{2CO}}_{3}}^{2-}+{\mathrm{OH}}^{-}$$

with proton donors on the left‐hand side and proton acceptors on the right‐hand side. The carbonate ion is counted twice because it is two protons below the reference level H_2_CO_3_.

The titration alkalinity, that is, excess of proton acceptors over donors with respect to carbonic acid, the reference level, is then defined as follows

(2) $$\mathrm{TA}={{\mathrm{HCO}}_{3}}^{-}+{{\text{2CO}}_{3}}^{2-}+{\mathrm{OH}}^{-}-H^{+}$$

Other acid–base systems can be included in the alkalinity expression. To this end, all chemical species in the solution have to be classified either as proton donor or acceptor relative to the zero level of protons for each acid–base system (Figure 1b). Dickson (1981) included fluoride, sulfate, borate, phosphate, silicate, ammonia, and hydrogen sulfide to eventually arrive at

$$\mathrm{TA}={{\mathrm{HCO}}_{3}}^{-}+{{\text{2CO}}_{3}}^{2-}+{\mathrm{OH}}^{-}+B\;{{\left(\mathrm{OH}\right)}_{4}}^{-}+{{\mathrm{HPO}}_{4}}^{2-}+2\;{{\mathrm{PO}}_{4}}^{3-}+H_{3}{{\mathrm{SiO}}_{4}}^{-}+{\mathrm{NH}}_{3}+{\mathrm{HS}}^{-}+2\;S^{2-}$$

(3) $$-H^{+}-\mathrm{HF}-{{\mathrm{HSO}}_{4}}^{-}-H_{3}{\mathrm{PO}}_{4}$$

This proton balance approach toward alkalinity allows an exact definition of alkalinity. Dickson (1981) focused on the quantification of alkalinity in seawater from titration data and, therefore, did not include the strong acids H_2_SO_4_ and HNO_3_ nor HNO_2_ and H_2_SiO_4_^2−^. However, these can easily be included using the same approach (Soetaert et al., 2007; Wolf‐Gladrow et al., 2007) and results in

$$\mathrm{TA}={{\mathrm{HCO}}_{3}}^{-}+{{\text{2CO}}_{3}}^{2-}+{\mathrm{OH}}^{-}+B\;{{\left(\mathrm{OH}\right)}_{4}}^{-}+{{\mathrm{HPO}}_{4}}^{2-}+2\;{{\mathrm{PO}}_{4}}^{3-}+H_{3}{{\mathrm{SiO}}_{4}}^{-}\;$$

(4) $$+2\;H_{2}{{\mathrm{SiO}}_{4}}^{2-}+{\mathrm{NH}}_{3}+{\mathrm{HS}}^{-}+2\;S^{2-}-H^{+}-\mathrm{HF}–{{\mathrm{HSO}}_{4}}^{-}-2\;H_{2}{\mathrm{SO}}_{4}-H_{3}{\mathrm{PO}}_{4}–{\mathrm{HNO}}_{2}–{\mathrm{HNO}}_{3}$$

where H_2_SO_4_ and HNO_3_ are zero for the pH values >0.

The titration alkalinity definition of Dickson (1981) is fully consistent with the conservation equation for total hydrogen ions (TOTH) of Morel and Hering (1993), which is also based on a proton (mass) balance (supporting information S1). Specifically, TA = −TOTH when the components chosen are the reference level species at pH = 4.5.

### 2.2 Charge Balance Alkalinity

Electrolyte solutions, including seawater, obey the electroneutrality condition, that is, the sum of negative and positive charges balances at the macroscale (Boudreau et al., 2004; Soetaert et al., 2007; Wolf‐Gladrow et al., 2007). For seawater, we thus have to balance the sum of cation concentrations

(5a) $${\mathrm{Na}}^{+}+2\;{\mathrm{Mg}}^{2+}+2\;{\mathrm{Ca}}^{2+}+K^{+}+2\;{\mathrm{Sr}}^{2+}+\left(\ldots \ldots \right)+{{\mathrm{NH}}_{4}}^{+}+H^{+}$$

with the sum of anion concentrations

(5b) $${\mathrm{Cl}}^{-}+{\mathrm{Br}}^{-}+\left(\ldots ..\right)+{{\mathrm{HCO}}_{3}}^{-}+{{\text{2CO}}_{3}}^{2-}+{\mathrm{OH}}^{-}+B\;{{\left(\mathrm{OH}\right)}_{4}}^{-}+H_{2}{{\mathrm{PO}}_{4}}^{-}+2\;{{\mathrm{HPO}}_{4}}^{2-}+3\;{{\mathrm{PO}}_{4}}^{3-}+H_{3}{{\mathrm{SiO}}_{4}}^{-}+2\;H_{2}{{\mathrm{SiO}}_{4}}^{2-}+{\mathrm{HS}}^{-}+2\;S^{2-}+F^{-}+{{\mathrm{HSO}}_{4}}^{-}+2\;{{\mathrm{SO}}_{4}}^{2-}+{{\mathrm{NO}}_{2}}^{-}+{{\mathrm{NO}}_{3}}^{-}$$

where the ellipses (…) stand for additional ions in solution. In charge conservation equations, ions are multiplied with their charge. This equation can be rearranged into a part that is conservative and a part that is not and involves species exchanging protons. Accordingly, when ignoring minor conservative species (…), the sum of strong base cations minus the sum of strong acid anions (i.e., excess positive charge of conservative ions)

(6a) $$\mathrm{EPC}={\mathrm{Na}}^{+}+2\;{\mathrm{Mg}}^{2+}+2\;{\mathrm{Ca}}^{2+}+K^{+}+2\;{\mathrm{Sr}}^{2+}-{\mathrm{Cl}}^{-}-{\mathrm{Br}}^{-}$$

should balance the excess negative charge (ENC) of nonconservative ions that are involved in proton exchange reactions

(6b) $$\mathrm{ENC}={{\mathrm{HCO}}_{3}}^{-}+{{\text{2CO}}_{3}}^{2-}+{\mathrm{OH}}^{-}+B\;{{\left(\mathrm{OH}\right)}_{4}}^{-}+H_{2}{{\mathrm{PO}}_{4}}^{-}+2\;{{\mathrm{HPO}}_{4}}^{2-}+3\;{{\mathrm{PO}}_{4}}^{3-}+H_{3}{{\mathrm{SiO}}_{4}}^{-}+.2\;H_{2}{{\mathrm{SiO}}_{4}}^{2-}+{\mathrm{HS}}^{-}+2\;S^{2-}+F^{-}+{{\mathrm{HSO}}_{4}}^{-}+2\;{{\mathrm{SO}}_{4}}^{2-}+{{\mathrm{NO}}_{2}}^{-}+{{\mathrm{NO}}_{3}}^{-}-{{\mathrm{NH}}_{4}}^{+}-H^{+}$$

This excess negative charge (Soetaert et al., 2007) is also known as CBA. Hence, CBA is defined as the sum of nonconservative ions involved in proton exchange reactions that account for the difference between the sum of conservative cations and anions.

CBA (Equation 6b) and TA (Equation 4) are linked via

(7) $$\mathrm{TA}=\mathrm{CBA}+\sum {\mathrm{NH}}_{3}-\sum {\mathrm{NO}}_{3}-\sum {\mathrm{NO}}_{2}-\sum {\mathrm{PO}}_{4}–2\sum {\mathrm{SO}}_{4}-\sum F$$

where ∑NH_3_ = NH_3_ + NH_4_^+^, ∑NO_3_ = NO_3_^−^ + HNO_3_, ∑NO_2_ = NO_2_^−^ + HNO_2_, ∑PO_4_ = H_3_PO_4_ + H_2_PO_4_^−^ + HPO_4_^2−^ + PO_4_^3−^, ∑SO_4_ = H_2_SO_4_ + HSO_4_^−^ + SO_4_^2−^ and ∑F = HF + F^−^ are the total concentrations of ammonia, nitrate, nitrite, phosphate, sulfate, and fluoride, respectively. This difference between titration and charge balance alkalinity is due to the charge of components at the reference pH level of 4.5. At pH 4.5, ammonia is present as ammonium (+1), while nitrate, nitrite, fluoride, and phosphate have an overall charge of −1 and sulfate is present as SO_4_^2−^ with charge −2 (Figure 1c). Accordingly, dissolved inorganic carbon does not appear in Equation 7 because it is present as the uncharged carbon dioxide at pH 4.5. In other words, the difference between CBA and TA is caused by components for which the species used as zero proton level are charged (e.g., H_2_PO_4_^−^ is the zero‐proton level for phosphate; Dickson, 1981).

Wolf‐Gladrow et al. (2007) introduced the explicitly conservative expression of total alkalinity (TA_ec_) that relates the sum of conservative cations and anions (i.e., excess positive charge; Equation 6a) and total concentrations of ammonia, nitrate, nitrite, phosphate, sulfate, and fluoride with titration alkalinity (TA)

(8) $${\mathrm{TA}}_{\mathrm{ec}}={\mathrm{Na}}^{+}+2\;{\mathrm{Mg}}^{2+}+2\;{\mathrm{Ca}}^{2+}+K^{+}+2\;{\mathrm{Sr}}^{2+}-{\mathrm{Cl}}^{-}-{\mathrm{Br}}^{-}+\left(\ldots \right).+\sum {\mathrm{NH}}_{3}-\sum {\mathrm{NO}}_{3}-\sum {\mathrm{NO}}_{2}-\sum {\mathrm{PO}}_{4}–2\sum {\mathrm{SO}}_{4}-\sum F$$

or alternatively formulated: TA_ec_ = EPC + TA − CBA.

This explicitly conservative form of alkalinity is equivalent to Dickson's expression (as EPC‐CBA = 0) for titration alkalinity (Equation 4), but each single term is conservative to proton exchange and pressure and temperature changes (Wolf‐Gladrow et al., 2007). Since charge balance alkalinity is directly related to the difference between conservative cations and anions, it is evident that CBA, and thus, also TA covary with salinity. Alkalinity and salinity are both affected to the same degree by processes that dilute or concentrate seawater, such as precipitation, evaporation, and melting or formation of ice.

### 2.3 Alternative Alkalinity and Related Expressions

The titration and charge balance alkalinity expressions (Equations 4 and 6b) are well defined and traceable to the use of a proton or charge balance (supporting information S1), but alternative expressions are often used. There are a number of reasons for this. One, alkalinity and related concepts are used in multiple disciplines (e.g., chemistry, medicine, environmental engineering, ecology, geology, hydrology, limnology, and oceanography) with their own specific scientific traditions and terminology. Two, although many species are included in the formal definition of alkalinity in seawater, a few of these dominate by far and most others can be ignored as a first‐order approximation. Carbonate alkalinity (CA = HCO_3_^−^ + 2CO_3_^2−^) typically accounts for >95% of the total alkalinity in the ocean. Many studies (e.g., Broecker & Peng, 1982) use a simple form of alkalinity including only water and carbonate alkalinity terms (Equation 2). In seawater, a slightly more accurate expression is obtained when borate alkalinity is included as well. Zeebe and Wolf‐Gladrow (2001) termed this alkalinity for most practical purposes (PA_ZW‐G_)

(9) $${\mathrm{PA}}_{\mathrm{ZW}-G}={{\mathrm{HCO}}_{3}}^{-}+{{\text{2CO}}_{3}}^{2-}+B\;{{\left(\mathrm{OH}\right)}_{4}}^{-}+{\mathrm{OH}}^{-}-H^{+}=\mathrm{CA}+\text{borate alkalinity}+\text{water alkalinity}.$$

PA_ZW‐G_ is often used interchangeably with TA because it typically represents >99% of total alkalinity in oxygenated ocean surface waters. In anoxic waters and pore waters of marine sediments in which metabolites (ammonia, phosphate, sulfide, and silicate) have accumulated, some of these are then included in the operational definition of alkalinity for that system (Ben‐Yaakov, 1973; Boudreau & Canfield, 1993; Hiscock & Millero, 2006). Finally, a major reason for alternative alkalinity expressions relates to application of the measurable property TA to biogeochemical processes that impact CBA because of electroneutrality constraints.

#### 2.3.1 Use of Titration Alkalinity as Proxy for Charge Balance Alkalinity

Charge balance and titration alkalinity differ by the total amounts of nitrite, nitrate, ammonia, phosphate, sulfate, and fluoride (Equation 7; Soetaert et al., 2007; Wolf‐Gladrow et al., 2007). Processes such as primary production, organic matter degradation, and nitrification involve these components and thus potentially impact alkalinity. Brewer and Goldman (1976) and Goldman and Brewer (1980) documented increases in alkalinity due to nitrate and phosphate uptake and a decrease in alkalinity due to ammonium uptake (see section 4). These alkalinity changes (ΔTA) due to biological consumption or production processes have to be taken into account when using measured TA values for quantification of calcium carbonate precipitation or dissolution. Specifically, Brewer et al. (1975) introduced the potential alkalinity change (ΔP.A.) as a measure of calcite formation/dissolution

(10) $$\mathrm{\Delta P}.A.=\mathrm{\Delta TA}+\Delta \sum {\mathrm{NO}}_{3}+\Delta \sum {\mathrm{PO}}_{4}.$$

By comparing Equations 10 and 7, it is clear that potential alkalinity change is a proxy for CBA change based on measured nitrate, phosphate, and TA. However, only changes in nitrate and phosphate due to biological processes should be considered, not those due to physical mixing (Huang et al., 2015). Similarly, Kanamori and Ikegami (1982) identified the need to include nitrate, phosphate, and sulfate when using measured TA for calculating alkalinity changes due to calcium carbonate dynamics. Including nitrate, phosphate, and sulfate contributions in Redfield proportions would result in the following expression for potential alkalinity (P.A.)

(11) $$P.A.=\mathrm{TA}+a*\;\sum {\mathrm{NO}}_{3}$$

where *a* varies from 1.06 (Chen, 1978) and 1.26 (Kanamori & Ikegami, 1982) to 1.36 (Wolf‐Gladrow et al., 2007), depending on the Redfield ratios considered.

The Alk* tracer (Carter et al., 2014) combines the potential alkalinity (Equation 11 with *a* = 1.26) with salinity normalization to single out the effect of calcium carbonate dynamics on alkalinity. Similarly, Feely et al. (2002) introduced the TA* tracer which expresses the change in TA due to calcium carbonate dynamics: TA* = 0.5 (TA_s_‐TA^o^_s_) + 0.63*(0.0941 AOU), where TA_s_ and TA^o^_s_ are the measured and preformed salinity‐normalized TA, respectively, and AOU is the apparent oxygen utilization, introduced to correct for charges generated during organic matter dynamics. Finally, ecologists studying calcification by benthic communities (coral reefs and bivalves) often employ the alkalinity anomaly technique (Chisholm & Gattuso, 1991; Gazeau et al., 2015; Kinsey, 1978), which involves measurement of TA and correcting it with ammonium, nitrate, and phosphate for obtaining calcium carbonate dynamics.

#### 2.3.2 Organic Alkalinity

While oceanographers usually measure TA and introduce empirical corrections to arrive at the CBA needed for quantitative applications (Equations 10 and 11), freshwater scientists studying soft natural waters have to use a charge balance of the major conservative ions because of an important contribution of organic acids (Hemond, 1990). Contrary to the inorganic acid–base species discussed before, dissolved organic compounds comprise a complex, heterogeneous group, which poses a challenge on classifying its acid–base properties and quantifying their contribution to TA (Hu, 2020). The composition and thus acid–base properties of dissolved organic compounds depend on whether these compounds are derived from locally produced organic matter or transported from adjacent terrestrial ecosystems (Leenheer & Croué, 2003). Phytoplankton‐derived dissolved organic compounds are found to have two distinct proton binding sites with pK values of 4.4–4.9 and 6.1–6.9, respectively (Ko et al., 2016). In contrast, terrestrially derived organic matter is dominated by humic substances. These comprise a much wider range of proton binding sites, often described by carboxyl and phenolic groups having average pK values of about 3.7 ± 2.4 and about 12.5 ± 1.8, respectively (Perdue et al., 1984). This wide range in pK values implies that at least part of the dissolved organic compounds, either of autochthonous or allochthonous origin, acts as proton acceptor at pK 4.5, thus contributing to TA despite being absent in Equation 4.

Substantial contributions of organic alkalinity to TA have been found in laboratory incubations (Ko et al., 2016), estuaries (Cai et al., 1998), sediment pore waters (Łukawska‐Matuszewska, 2016; Łukawska‐Matuszewska et al., 2018), coastal waters receiving high terrestrial inputs, like the Baltic Sea (Hammer et al., 2017; Kuliński et al., 2014) or salt marsh‐influenced coastal waters (Song et al., 2020), and ocean waters (Fong & Dickson, 2019). Organic alkalinity is normally assessed by difference, that is, carbonate alkalinity is derived from two out of three other measurable parameters in the CO_2_‐H_2_O system (pH, DIC, or pCO_2_), and organic alkalinity is calculated as the difference between TA measured and calculated from the contributions of the inorganic species following Equation 3. This method relies on at least one parameter (pH or pCO_2_) which value is affected by the presence of organic compounds and, therefore, does not allow for an exact value of organic alkalinity. Back‐titration methods to directly quantify organic alkalinity are used by others (Cai et al., 1998; Hernandez‐Ayon et al., 2007; Muller & Bleie, 2008; Yang et al., 2015), showing no clear correlation with organic alkalinity estimated by difference (Song et al., 2020). Alternatively, chemical equilibrium models describing proton binding to humic substances, which are well‐known in the freshwater community (Kinniburgh et al., 1999), can be coupled to inorganic carbonate system calculations (Ulfsbo et al., 2015).

#### 2.3.3 Acid Neutralizing Capacity

Although not often used in oceanography, the term acid neutralizing capacity (ANC) is closely linked to titration alkalinity (TA). The ANC of a solution to the carbonic acid equivalent point of a titration is fully consistent with the Dickson (1981) definition of TA (Stumm & Morgan, 1981; Weber & Stumm, 1963). Other equivalence points are termed p‐ alkalinity (phenolphtalein endpoint of titration, corresponding to the proton balance of Equation 1.13 in supporting information S1) and caustic alkalinity, the reverse of acidity, with the proton balance: TOTH = OH^−^ − H^+^ − 2 H_2_CO_3_ − HCO_3_^−^ (Stumm & Morgan, 1981). Theoretically, one can use any expression for alkalinity to solve the CO_2_‐H_2_O system as long it is properly defined.

Some researchers distinguish between TA and ANC whether water samples are filtered or not, respectively (Asuero & Michałowski, 2011; Michałowski & Asuero, 2012). The chemical model underlying Dickson's TA only includes homogeneous reactions in solution and ignores proton exchange with particles and organisms. This implies that water samples for alkalinity should be filtered before titration because of potential proton exchange with the surface of phytoplankton, bacteria, and inorganic particles and the dissolution of suspended particulate inorganic carbon (Kim et al., 2006), and dedicated filtration methods have been developed (Bockman & Dickson, 2014). However, differences between filtered and unfiltered samples are often negligible (open ocean, Chanson & Millero, 2007; coastal systems, Hagens et al., 2015) but might be substantial in experimental systems with high densities of organisms or particles.

## 3 Buffering and Sensitivity Factors

Seawater is a solution with multiple weak acids and bases in contact with both the atmosphere and sediments containing minerals that have the potential to react when solution composition or physical conditions change. Seawater is consequently well buffered, that is, able to resist changes by transferring protons. The response of a chemical equilibrium system to a perturbation follows the principle of le Chatelier. The original statement of Henry Louis le Chatelier (1884) *“Tout système en équilibre chimique stable soumis à l*'*influence d*'*une cause extérieure qui tend à faire varier soit sa température, soit sa condensation (pression, concentration, nombre de molécules dans l*'*unité de volume) dans sa totalité ou seulement dans quelques‐unes de ses parties, ne peut éprouver que des modifications intérieures, qui, si elles se produisaient seules, amèneraient un changement de température ou de condensation de signe contraire à celui résultant de la cause extérieure.”* is often rephrased as follows: whenever a system in equilibrium is disturbed by changing the conditions, the positions of the equilibria shift in such a way that the effect of the change will be moderated.

In this section, we discuss the sensitivity and resistance of ocean chemistry to changes. It is instructive to distinguish between homogeneous reactions in solution and heterogeneous buffering involving interactions with particles (e.g., dissolution or precipitation of carbonate minerals modifying alkalinity). Homogeneous buffering takes place nearly instantaneously and is most relevant for quantifying and understanding the impact of biogeochemical processes on pH on short (hour‐days) timescales (Egleston et al., 2010; Frankignoulle, 1994; Soetaert et al., 2007). Heterogeneous buffering reactions may involve very long time scales (months to millions of years) and will be discussed in section 5.

### 3.1 Buffer Capacity Systematics

Although the buffer capacity of seawater and its role in earth system science has been recognized in the first part of the twentieth century (Mitchell & Rakestraw, 1933; Thompson & Bonnar, 1931) and mathematical tools to quantify buffer efficiency have been developed a century ago (Koppel & Spiro, 1914; van Slyke, 1922), quantitative treatments of seawater buffering have historically received little attention, except for the homogeneous Revelle factor (Revelle & Suess, 1957) and the acid–base buffer capacity (van Slyke, 1922; Weber & Stumm, 1963). The acid–base buffer value β was originally defined for biological fluids by Koppel and Spiro (1914) but is commonly attributed to van Slyke (1922)

(12) $$\beta =\frac{-dC_{a}}{\mathrm{dpH}}$$

where C_a_ is the quantity of acid added to a solution. For seawater, TA is substituted for C_a_ and partial derivatives are used to indicate that other properties are kept constant during the titration

(13) $$\beta =\frac{\partial \mathrm{TA}}{\partial \mathrm{pH}}$$

The buffer value β is always positive because every solution resists pH change according to the principle of le Chatelier. It is based on pH rather than proton concentrations because of historical reasons and laboratory procedures.

The Revelle factor (R; Revelle & Suess, 1957; Bolin & Eriksson, 1959; Sundquist et al., 1979) expresses the sensitivity of pCO_2_ to changes in DIC

(14) $$R=\frac{\partial \text{lnpC}O_{2}}{\partial \text{lnDIC}}=\frac{\mathrm{DIC}}{\mathrm{pC}O_{2}}\left(\frac{\partial \mathrm{pC}O_{2}}{\partial \mathrm{DIC}}\right)$$

where use is made of the property $\partial \ln x=\frac{1}{x}\partial x.$ This Revelle factor is limited to homogeneous systems, because the partial derivatives indicate that other variables such as alkalinity are kept constant. Sundquist and Plummer (1981) extended the homogeneous Revelle factor to allow for changes in alkalinity (e.g., due to calcification/dissolution)

(15) $$\text{Rtot}=\frac{\mathrm{DIC}}{\mathrm{pC}O_{2}}\left(\frac{\mathrm{dpC}O_{2}}{\text{dDIC}}\right)=\;\frac{\mathrm{DIC}}{\mathrm{pC}O_{2}}\left[\;{\left(\frac{\partial \mathrm{pC}O_{2}}{\partial \mathrm{DIC}}\right)}_{\mathrm{TA}}+{\left(\frac{\partial \mathrm{pC}O_{2}}{\partial \mathrm{TA}}\right)}_{\mathrm{DIC}}\;\cdot \frac{\mathrm{dTA}}{\text{dDIC}}\right]$$

In seawater, the term ${\left(\frac{\partial \mathrm{pC}O_{2}}{\partial \mathrm{TA}}\right)}_{\mathrm{DIC}}$ is negative, while $\frac{\mathrm{dTA}}{\text{dDIC}}$ varies from zero (no TA change) to 2 when all changes in DIC are due to calcium carbonate dissolution; heterogeneous buffering thus lowers the Revelle factor (i.e., ocean buffering is larger when carbonate minerals are involved).

The Revelle and acid–base buffer factors are just two out of many ways to quantify the response of seawater to changes and some additional (chemical) buffer factors have been proposed (e.g., Frankignoulle, 1994; Frankignoulle et al., 1994; Egleston et al., 2010; Hagens & Middelburg, 2016a; Table 1). However, there are multiple inconsistencies in terminology and their relationships are not clear (Table 1). To clarify matters and link the various buffer capacities and factors in the literature, we present a systematic treatment involving partial derivatives as sensitivities. While the application of Le Chatelier's principle is straightforward for simple systems, it becomes difficult to predict the response of individual reactions when multiple reactions sharing ions are involved (Fishtik et al., 1995) and a sensitivity analysis is then useful. Sensitivities are also known as chemical buffer factors (Egleston et al., 2010; Frankignoulle, 1994; Soetaert et al., 2007). Sensitivities express the rate of change of output quantities (Y) in terms of input quantities (X), that is, their partial derivatives (Morel et al., 1976; Smith & Missen, 2003). The (first order) sensitivity coefficient (Smith & Missen, 2003) or interaction capacity (Morel et al., 1976) is defined as follows

(16) $$S_{Y,X}=\frac{\partial Y}{\partial X}$$

which can be normalized using logarithms so that the percentage change in output can be directly linked to percentage change in input (interaction intensity values [Morel et al., 1976] or normalized first‐order sensitivities [Smith & Missen, 2003])

(17) $$S_{Y,X}^{\mathrm{no}r}=\frac{\partial \mathrm{lnY}}{\partial \mathrm{lnX}}$$

In the case of pH during an acid titration of TA, the sensitivity is the inverse of the well‐known acid–base buffer capacity (β; Equation 13).

(18) $$S_{\mathrm{pH},\mathrm{TA}}=\frac{\partial \mathrm{pH}}{\partial \mathrm{TA}}=\beta^{-1}$$

**Table 1:** Overview of sensitivities of the Ocean Carbon System $\left(\frac{\partial \text{response}}{\partial \text{driver}}\right)$ and Their Relations to Buffering Quantities in the Literature

Driver	Response	Sensitivity factor	Symbol	Name	Reference
TA	pH	$\left(\frac{\partial \mathrm{pH}}{\partial \mathrm{TA}}\right)$	−Φ_*H*_ $\beta_{\mathrm{pH}}^{-1}$	Chemical buffer factor Inverse of Buffer capacity	Frankignoulle (1994) van Slyke (1922)
	lnH	$\left(\frac{\partial \mathrm{lnH}}{\partial \mathrm{TA}}\right)$	$\beta_{\mathrm{TA}}^{-1}$		Egleston et al. (2010)
	H	$\left(\frac{\partial H}{\partial \mathrm{TA}}\right)$	$-\beta_{H}^{-1}$	Negative inverse of Proton concentration buffer factor	Hofmann et al. (2010a & 2010b)
	pCO_2_	$\left(\frac{\partial pCO_{2}}{\partial \mathrm{TA}}\right)$	−Π_*H*_	Buffer factor	Frankignoulle (1994)
	lnpCO_2_	$\left(\frac{\partial pCO_{2}}{\partial \mathrm{TA}}\right)\frac{\mathrm{TA}}{pCO_{2}}\;\text{or}$ $\left(\frac{\partial \ln pCO_{2}}{\partial \text{lnTA}}\right)$	*γ*_*TA*_	Alkalinity factor	Sarmiento and Gruber (2006)
	lnCO_2_	$\left(\frac{\partial \mathrm{lnC}O_{2}}{\partial \mathrm{TA}}\right)$	$\gamma_{\mathrm{TA}}^{-1}$		Egleston et al. (2010)
	CO_2_	$\frac{1}{K_{0}}\left(\frac{\partial CO_{2}}{\partial \mathrm{TA}}\right)$	−Π_*H*_		Frankignoulle (1994)
	lnCO_3_^2−^	$\left(\frac{\partial \mathrm{lnC}O_{3}^{2-}}{\partial \mathrm{TA}}\right)$	$\omega_{\mathrm{TA}}^{-1}$		Egleston et al. (2010)
DIC	pH	$\left(\frac{\partial \mathrm{pH}}{\partial \mathrm{DIC}}\right)$	Φ $\beta_{CO_{2}}^{-1}$		Frankignoulle (1994) and Weber & Stumm (1963)
	lnH	$\left(\frac{\partial \mathrm{lnH}}{\partial \mathrm{DIC}}\right)$	$\beta_{\mathrm{DIC}}^{-1}$		Egleston et al. (2010)
	pCO_2_	$\left(\frac{\partial pCO_{2}}{\partial \mathrm{DIC}}\right)$	Π_*D*_		Frankignoulle (1994)
	lnpCO_2_	$\left(\frac{\partial pCO_{2}}{\partial \mathrm{DIC}}\right)\frac{\mathrm{DIC}}{pCO_{2}}\;\text{or}$ $\left(\frac{\partial \ln pCO_{2}}{\partial \text{lnDIC}}\right)$	R *B*_*hom*_ *β*_*D*_ *γ*_*DIC*_	Homogeneous buffer factor or Revelle factor	Bolin and Eriksson (1959), Sundquist et al. (1979), Frankignoulle (1994), and Sarmiento and Gruber (2006)
	lnCO_2_	$\left(\frac{\partial \mathrm{lnC}O_{2}}{\partial \mathrm{DIC}}\right)$	$\gamma_{\mathrm{DIC}}^{-1}$		Egleston et al. (2010)
	TA	$\left(\frac{\partial \mathrm{TA}}{\partial \mathrm{DIC}}\right)$	Q	Isocapnic quotient	Humphreys et al. (2018)
	lnCO_3_^2−^	$\left(\frac{\partial \mathrm{lnC}O_{3}^{2-}}{\partial \mathrm{DIC}}\right)$	$\omega_{\mathrm{DIC}}^{-1}$		Egleston et al. (2010)
pCO_2_	pH	$\left(\frac{\partial \mathrm{pH}}{\partial pCO_{2}}\right)$	Φ_*D*_		Frankignoulle (1994)
	lnH	$\left(\frac{\partial \mathrm{lnH}}{\partial \ln pCO_{2}}\right)$	*H*^−1^		Omta et al. (2010)
	lnCO_3_^2−^	$\left(\frac{\partial CO_{3}^{2-}}{\partial pCO_{2}}\right)\frac{pCO_{2}}{CO_{3}^{2-}}\;\text{or}$ $\left(\frac{\partial \mathrm{lnC}O_{3}^{2-}}{\partial \ln pCO_{2}}\right)$	$\beta_{C}^{-1}O^{-1}$		Frankignoulle (1994) and Omta et al. (2010)

*Note*. Based on the Hagens and Middelburg (2016a) approach, supporting information S2 explicitly links the various sensitivities.

The use of both sensitivities (=tendency to change), in which the cause of change is in the denominator and the resulting change is in the numerator (e.g., $\frac{\partial \mathrm{pH}}{\partial \mathrm{TA}}),$ and buffering capacities (=resistance to change), which are just the inverse (e.g., $\frac{\partial \mathrm{TA}}{\partial \mathrm{pH}})$ is one of the reasons for confusion in the literature. Buffer capacity β expresses the ability to resist changes and is normally presented as

(19) $$\beta_{\mathrm{pH}}={\left(\frac{\partial \mathrm{pH}}{\partial \mathrm{TA}}\right)}^{-1}$$

(Middelburg, 2019; Morel & Hering, 1993; Stumm & Morgan, 1981). The Revelle factor (R; Equation 14) is a (normalized) sensitivity factor.

Another cause of inconsistencies among studies relates to the use of pH, ln[H^+^], or [H^+^]. The original buffer factor β is based on pH (Equations 13 and 19), but Egleston et al. (2010) and Hofmann, Middelburg, et al. (2010) presented definitions based on the natural logarithm of proton concentrations and proton concentrations, respectively

(20, 21) $$\beta_{\mathrm{TA}}={\left(\frac{\partial \mathrm{lnH}}{\partial \mathrm{TA}}\right)}^{-1}\;\text{and}\;\beta_{H}={\left(\frac{\partial H}{\partial \mathrm{TA}}\right)}^{-1}$$

While β_pH_ values are always positive, β_H_ and β_TA_ are negative. Note that these buffer factors have been defined as inverse of sensitivity factors. The interchangeable and inconsistent use of the terms buffer capacity, intensity, and index for *β* is another cause of confusion. Analytical chemists favor the term buffer capacity, geochemists prefer buffer intensity and engineers use the term buffer index (Urbansky & Schock, 2000). Others distinguish between buffer intensity for the actual value at a certain pH (the differential) and buffer capacity for the integral over a distinct range (Chiriac & Balea, 1997).

Using a first‐order sensitivity approach the response in Y due to changes in the drivers TA, DIC, T, S, and any other property X can be described as follows

(22) $$\mathrm{dY}=\left(\frac{\partial Y}{\partial \mathrm{TA}}\right)\mathrm{dTA}+\left(\frac{\partial Y}{\partial \mathrm{DIC}}\right)\text{dDIC}+\left(\frac{\partial Y}{\partial T}\right)\mathrm{dT}+\left(\frac{\partial Y}{\partial S}\right)\mathrm{dS}+\left(\frac{\partial Y}{\partial X}\right)\mathrm{dX}$$

where the partial derivatives imply that the other factors are constant. These and other sensitivities have been used and named in the literature, either in this particular, a normalized or similar form (Table 1). Sometimes a different name is used for the same sensitivity or the same name is used for different sensitivities. For instance, Sarmiento and Gruber (2006) define their alkalinity factor (*γ*_*TA*_) as

(23) $$\gamma_{\mathrm{TA}}=\left(\frac{\partial pCO_{2}}{\partial \mathrm{TA}}\right)\frac{\mathrm{TA}}{pCO_{2}}=\left(\frac{\partial \ln pCO_{2}}{\partial \text{lnTA}}\right)$$

while Egleston et al. (2010) use the same symbol for a different sensitivity:

(24) $$\gamma_{\mathrm{TA}}=\left(\frac{\partial \mathrm{TA}}{\partial \mathrm{lnC}O_{2}}\right)$$

Similarly, the Revelle factor (R; Equation 14) is also known as homogeneous buffer factor B_hom_ (Sundquist et al., 1979), *β*_*D*_ (Frankignoulle, 1994), and *γ*_*DIC*_ (Sarmiento & Gruber, 2006). Moreover, it relates to DIC/*γ*_*DIC*_ using the *γ*_*DIC*_ definition of Egleston et al. (2010), which is again different from that of Sarmiento and Gruber (2006). Most of the sensitivities in Table 1 can be explicitly linked (Frankignoulle, 1994; Hagens & Middelburg, 2016a) as documented in supporting information S2. *To prevent further confusion and propagation of inconsistencies, we propose to explicitly add the term sensitivity to factors such as the Revelle sensitivity factor and restrict the use of buffer for its inverse, that is, the resistance to change. Consequently, we discourage the common use of the term buffer or chemical buffer factor for sensitivities*.

### 3.2 Sensitivity of Seawater

**Figure 2:**
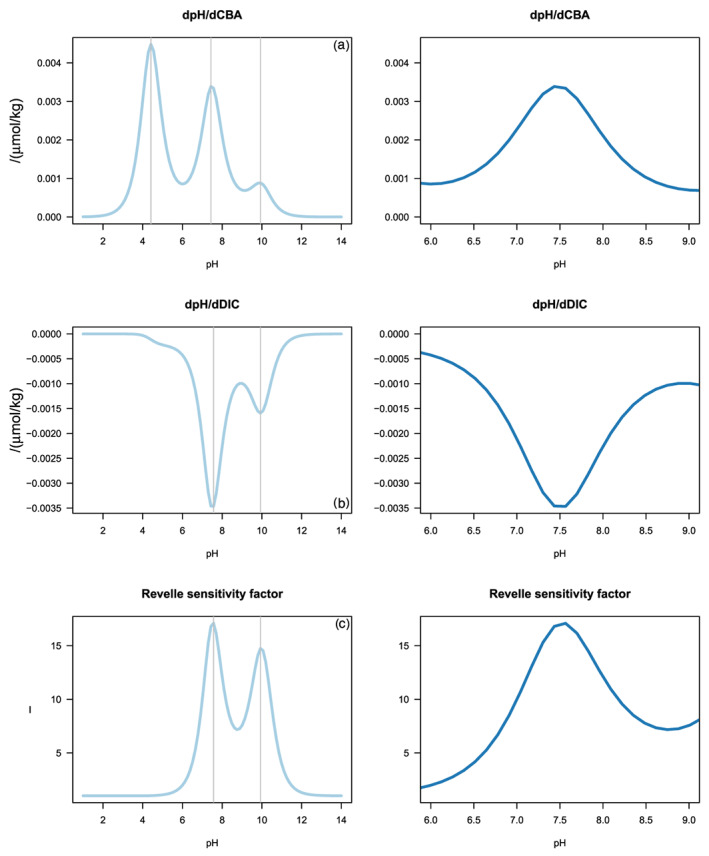
The sensitivities dpH/dCBA (a), dpH/dDIC (b), and the Revelle sensitivity factor (c) as function of pH. Left column over the entire pH range; right column focuses on the changes from pH 6 to 9. Vertical gray lines indicate maxima/minima. Details of the calculations are presented in the supporting information.

Most of the sensitivities presented above depend nonlinearly on the solution composition. Figure 2 shows the sensitivities of pH toward changes in CBA and DIC and of pCO_2_ toward a change in DIC (Revelle sensitivity factor) as a function of pH for average seawater. The carbonic acid system dominates the buffering capacity of seawater and these sensitivities thus show extrema related to the pK_1_ (≈5.9) and pK_2_ (≈9) values of carbonic acid in seawater. The sensitivity $\left(\frac{\partial \mathrm{pH}}{\partial \mathrm{CBA}}\right)$ shows maxima at pH values of about 4.5 and 7.5. The former is the proton reference level chosen at the well‐known inflection point of the alkalinity titration (Dickson, 1981), and the latter is consistent with the minor species theorem that it should be half way between pK_1_ and pK_2_ (Egleston et al., 2010; Morel & Hering, 1993). These sensitivities show minima close to the pK_1_ and pK_2_ values of carbonic acid in seawater, consistent with the well‐established concept that buffers are most efficient close to their pK value (Butler, 1964; Stumm & Morgan, 1981). At pH values of about 7.5, sensitivity toward addition of dissolved inorganic carbon $\left(\frac{\partial \mathrm{pH}}{\partial \mathrm{DIC}}\right),$ a weak acid, is similar to that of $\left(\frac{\partial \mathrm{pH}}{\partial \mathrm{CBA}}\right),$ addition of proton acceptors, but for the sign. However, seawater is more sensitive to CBA than to DIC changes below the pK_1_ of the carbonic acid system (pH ≈ 5.9). Conversely, the sensitivity toward DIC changes is larger than that due to CBA changes at pH > 7.5. This can be attributed to the number of protons released (Egleston et al., 2010). The Revelle sensitivity factor is very low at pH values below 6 and above 12 and shows maxima at pH values of about 7.5 and 10 and a minimum around the pK_2_ of the carbonic acid system (pH ≈ 9) because of the prominent role of the carbonate ion in buffering the carbon dioxide added (Gattuso & Hansson, 2011)

(25) $$H_{2}O+{\mathrm{CO}}_{2}+{{\mathrm{CO}}_{3}}^{2-}\Leftrightarrow 2\;{{\mathrm{HCO}}_{3}}^{-}$$

These and other seawater sensitivities have many applications, ranging from propagating uncertainties in the carbonic acid system (Orr et al., 2018), attributing changes in pCO_2_ to temperature, salinity, and other factors (Middelburg, 2019; Sarmiento & Gruber, 2006; Takahashi et al., 1993, 2014), understanding factors governing pH seasonality (Hagens & Middelburg, 2016b), and how these factors will change because of global warming and ocean acidification (Hagens & Middelburg, 2016a). For instance, Richier et al. (2018) showed that the CO_2_ sensitivity of phytoplankton correlates with the sensitivity $\left(\frac{\partial \mathrm{pH}}{\partial \mathrm{DIC}}\right)$ of seawater. This quantification of sensitivities is pivotal to understanding earth system functioning and the magnitude of climate feedbacks during times of global change. For instance, Frankignoulle et al. (1994) showed how the stoichiometry of carbon dioxide release during calcite precipitation would change with increasing atmospheric carbon dioxide levels. Multiple authors have shown that the seasonality of pH and pCO_2_ will increase due to elevated sensitivities induced by ocean acidification (Gallego et al., 2018; Hagens & Middelburg, 2016a; Kwiatkowski & Orr, 2018; Riebesell et al., 2009; Schulz & Riebesell, 2013). Seawater sensitivity analysis has also contributed to elucidating interactions among various factors perturbing seawater pH and pCO_2_ such as hypoxia (Cai et al., 2011, 2017; Hagens et al., 2015; Hagens & Middelburg, 2016a) and atmospheric deposition (Hagens et al., 2014). Finally, explicit quantification of sensitivities allows estimation of simple projections or retrodictions. For instance, the present‐day value for $\left(\frac{\partial \mathrm{pH}}{\partial \mathrm{pC}O_{2}}\right)$ is about −0.0011 (ppmv^−1^; Hagens & Middelburg, 2016a). Combining this sensitivity with global annual increases in pCO_2_ of 1.1 to 2.1 ppmv year^−1^ for the periods 1964–1975 and 2005–2014 generates annual ocean pH declines of about 0.0012 to 0.0023 units, similar to that observed (Dore et al., 2009). Similarly, using the sensitivity $\left(\frac{\partial \mathrm{DIC}}{\partial \mathrm{pC}O_{2}}\right)$ of about 0.51 (μM kg^−1^/ppmv), one would retrodict that the global ocean surface DIC would have increased by about 1 μM kg^−1^ year^−1^ from 1994 to 2007, consistent with observations by Gruber et al. (2019).

## 4 Biogeochemical Processes and Inorganic Carbon Dynamics

**Figure 3:**
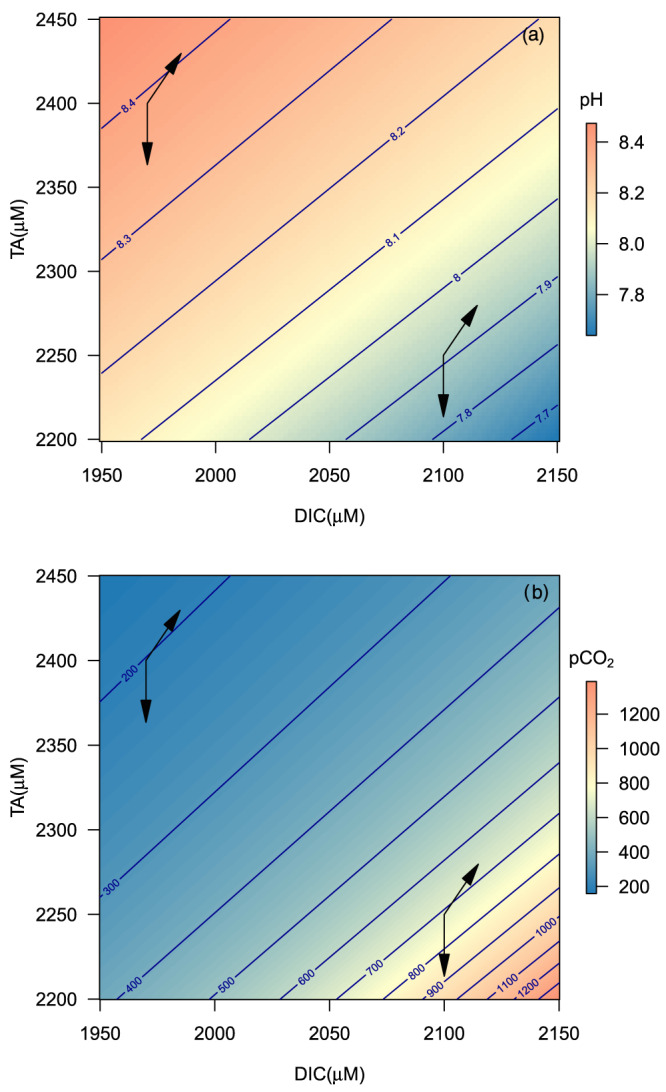
TA versus DIC plots (i.e., Deffeyes diagrams) showing the equilibrium pH at free scale (a) and pCO_2_ in μatm (b) as contours. Both graphs show vectors for the addition of protons (vertically downward) and dissolution of calcium carbonate (slope 2:1). Notice that the resulting change in pH and pCO_2_ for the same amount of calcite dissolved or acid added (same vector) differs because of differences in sensitivity (buffering). For instance, the ΔpH and ΔpCO_2_ for proton additions are −0.074 and +136 μatm, respectively, at low buffering (high DIC/TA ratio), and −0.037 and +20.9 μatm at high buffering (low DIC/TA ratio). Similarly, for the calcite dissolution vector, the ΔpH values are 0.022 and 0.013 and the ΔpCO_2_ values are −33.9 and −5.9 μatm for low and high buffering, respectively.

The marine carbon dioxide system is impacted by many biogeochemical processes: mineral dissolution and precipitation, organic matter production and respiration, and transfers of electrons, that is, redox processes (Middelburg, 2019; Soetaert et al., 2007; Wolf‐Gladrow et al., 2007). The impact of biogeochemical processes on pH and pCO_2_ is often analyzed graphically in the form of TA versus DIC plots with isolines for pH and pCO_2_ (Figure 3). The impact of biogeochemical processes on DIC, TA, or any of its constituents can be represented as a vector (Deffeyes, 1965). For instance, calcium carbonate dissolution results in the release of one unit DIC and two units of TA and the resulting vector on Figure 3 shows that it will cause an increase in pH and decrease in pCO_2_. However, for the very same process intensity (vector length and direction), the resulting change in pH and pCO_2_ is different because it depends on the initial conditions (i.e., the sensitivity of the system; section 3.1). Changes in pH and pCO_2_ are smaller in well buffered water with a high TA: DIC ratio, that is, low sensitivity (Figure 3).

Although this graphical approach is instructive, there is a need to quantify these changes to improve our predictive capabilities. Ben‐Yaakov (1973) recognized that a given reaction can change the pH of a solution by changing the total charge or by adding (or removing) an acid or base. Soetaert et al. (2007) elaborated this approach and showed that the effect of a single biogeochemical process on pH can be calculated from the product of the net charge exchanged during a biogeochemical process (Δcharge) and the sensitivity factor of seawater $\left(\frac{\partial \mathrm{pH}}{\partial \mathrm{CBA}}\right).$ Specifically, the instantaneous effect of a single process with intensity I_process_ (mol m^−3^ s^−1^) on pH can be calculated as follows

(26) $$\mathrm{dpH}\;=\;\Delta \text{charge}\cdot \left(\frac{\partial \mathrm{pH}}{\partial \mathrm{CBA}}\right)\cdot I_{\text{process}}$$

where Δcharge is the sum of the pH independent *ΔCBA* (Table 2) and the pH dependent charge (i.e., proton transfer) calculated from reaction stoichiometry and charge of acid–base systems (see Soetaert et al., 2007). Since both the sensitivity of seawater $\left(\frac{\partial \mathrm{pH}}{\partial \mathrm{CBA}}\right)$ and Δcharge depend on the pH, this equation shows that the effect of a specific biogeochemical process on pH also depends on pH. Hofmann et al. (2010a) and Middelburg (2019) presented similar approaches, but focused on proton rather than charge transfers and used the sensitivity factors $\left(\frac{\partial H}{\partial \mathrm{TA}}\right)\;$ and $\left(\frac{\partial \mathrm{pH}}{\partial \mathrm{TA}}\right),$ respectively.

**Table 2:** Biogeochemical Processes and Their Impact on Charge Balance Alkalinity (ΔCBA) and Titration Alkalinity (ΔTA) (After Soetaert et al., 2007)

Process	Reaction	ΔCBA	ΔTA
(R1) Aerobic Mineralization	(CH_2_O)(NH_3_)_n_(H_3_PO_4_)_p_ + O_2_ → CO_2_ + n NH_3_ + p H_3_PO_4_ + H_2_O	0	n ‐ p
(R2) Denitrification	(CH_2_O)(NH_3_)_n_(H_3_PO_4_)_p_ + 0.8 HNO_3_ → CO_2_ + n NH_3_ + p H_3_PO_4_ + 0.4 N_2_ + 1.4 H_2_O	0	0.8 + n − p
(R3) Mn‐Oxide Reduction	(CH_2_O)(NH_3_)_n_(H_3_PO_4_)_p_ + 2 MnO_2_ + 4H^+^ → CO_2_ + n NH_3_ + p H_3_PO_4_ + 2 Mn^2+^ + 3H_2_O	4	n − p + 4
(R4) Fe‐Oxide Reduction	(CH_2_O)(NH_3_)_n_(H_3_PO_4_)_p_ + 2 Fe_2_O_3_ + 8H^+^ → CO_2_ + n NH_3_ + p H_3_PO_4_ + 4 Fe^2+^ + 5H_2_O	8	n − p + 8
(R5) Sulfate Reduction	(CH_2_O)(NH_3_)_n_(H_3_PO_4_)_p_ + 0.5 H_2_SO_4_ → CO_2_ + n NH_3_ + p H_3_PO_4_ + 0.5 H_2_S + H_2_O	0	n − p + 1
(R6) Methanogenesis	(CH_2_O)(NH_3_)_n_(H_3_PO_4_)_p_ → 0.5 CO_2_ + n NH_3_ + p H_3_PO_4_ + 0.5 CH_4_ + H_2_O	0	n − p
(R7) Nitrification	NH_3_ + 2 O_2_ → HNO_3_ + H_2_O	0	−2
(R8) Annamox	NH_3_ + HNO_2_ → N_2_ + H_2_O	0	0
(R9) Aerobic Oxidation of Methane	CH_4_ + O_2_ → CO_2_ + 2 H_2_O	0	0
(R10) Anaerobic Oxidation of Methane	CH_4_ + H_2_SO_4_ → CO_2_ + H_2_S + 2 H_2_O	0	2
(R11) Calcite Precipitation	Ca^2+^ + CO_3_^2−^ → CaCO_3_	‐2	−2
(R12) Primary Production (Nitrate)	CO_2_ + n HNO_3_ + p H_3_PO_4_ + (1 + n) H_2_O → (CH_2_O)(NH_3_)_n_(H_3_PO_4_)_p_ + (1 + 2n) O_2_	0	p + n
(R13) Primary Production (Ammonium)	CO_2_ + n NH_3_ + p H_3_PO_4_ + H_2_O → (CH_2_O)(NH_3_)_n_(H_3_PO_4_)_p_ + O_2_	0	p − n
(R14) CO_2_ Emission to Air	CO_2_ → CO_2 (g)_	0	0
(R15) Proton Sorption	H^+^ → H^+^‐surface	1	1
(R15) Ammonium sorption	NH_4_^+^ → NH_4_^+^‐surface	1	0

*Note*. n = N/C ratio of organic matter and p = P/C ratio of organic matter.

**Figure 4:**
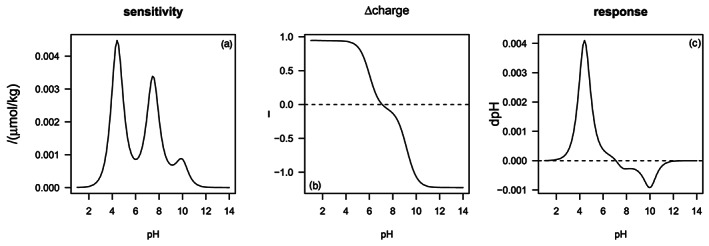
The response in pH due to denitrification as a function of pH. (a) The sensitivity factor dpH/dCBA of seawater, (b) the change in charge due to denitrification, and (c) the resulting change in pH as calculated by Equation 26. Details of the calculations are presented in the supporting information.

Figure 4 shows the impact of denitrification with Redfield organic matter (R2 in Table 2) on the Δcharge and pH. The process involves the production of DIC, ∑NH_3_, and ∑PO_4_, and the consumption of ∑NO_3._ Denitrification does not impact charge balance alkalinity (*ΔCBA* = 0). At pH < 4, nitrate is charged negatively, ammonium positively while DIC and ∑PO_4_ are present as uncharged carbonic and phosphoric acids, the Δcharge of the reaction is about +0.95. At pH > 10, nitrate is still charged negatively, while carbonate and phosphate ions dominate the DIC and ∑PO_4_ with the consequence that the Δcharge of the reaction is about −1.08 (Figure 4). At a pH of about 7, the Δcharge is zero because the positive charge due to nitrate consumption and ammonium production is compensated by the production of bicarbonate, the major species in the DIC pool at this pH, with contributions from carbonate, dihydrogenphosphate and hydrogenphosphate. The overall effect of denitrification on pH is obtained by multiplying the Δcharge and sensitivity at a specific pH value (Equation 26; Figure 4). Consequently, the pH increases at pH values below about 7 (Δcharge > 0), while it decreases pH at higher pH because the production of bicarbonate, carbonate and phosphate ions outcompetes the consumption of nitrate and production of ammonium (Δcharge < 0). Thus, Δcharge defines the direction of the pH change (i.e., increase or decrease), while the sensitivity determines the magnitude of the response.

**Figure 5:**
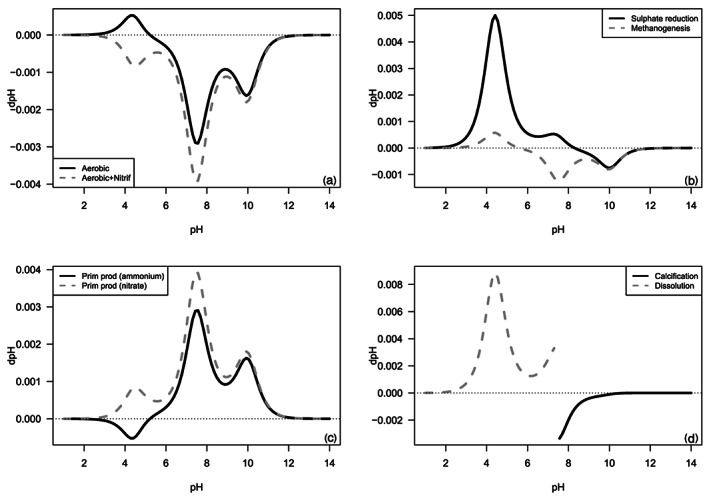
The impact of biogeochemical processes on pH. (a) The change in pH for aerobic mineralization with and without nitrification. (b) The change in pH due to sulfate reduction and methanogenesis. (c) The change in pH due to primary production based on ammonium or nitrate. (d) The change in pH due to calcification and calcium carbonate dissolution. Note the break at the pH corresponding to calcium carbonate equilibrium (modified from Soetaert et al., 2007). Details of the calculations are presented in the supporting information.

Soetaert et al. (2007) presented a full list of processes and how they impact pH over the full pH range; here we focus on aerobic and anaerobic mineralization, primary production, and calcium carbonate production and dissolution (Table 2). Figure 5 shows that the overall impact of a biogeochemical process on pH is a highly nonlinear function with multiple processes crossing the zero‐pH‐change line, implying that the direction (sign of Δcharge) and magnitude (sensitivity times Δcharge) of pH change depend on the initial conditions. For instance, calcium carbonate dissolution always increases the pH but the response depends on the sensitivity factor, that is, on the initial conditions, consistent with the graphical approaches presented above (Figure 3). Aerobic respiration (R1) and methanogenesis (R6) increase pH at pH < 5.2 and 5.6, respectively (Soetaert et al., 2007), because the production of ammonium is not compensated by sufficient production of anions (bicarbonate and phosphate anions). At higher pH, aerobic respiration and methanogenesis decrease pH because of bicarbonate, carbonate, and phosphate ion production (Figure 5). Aerobic respiration accompanied by nitrification (oxidation of ammonium to nitrate, R 7) always results in a pH decrease (Figure 5) because there is no positive charge produced (Soetaert et al., 2007). Similarly, primary production based on nitrate always results in pH increase, while regenerated production based on ammonium results in pH decrease at low pH (ammonium uptake is compensated by proton release for electroneutrality; Soetaert et al., 2007; Wolf‐Gladrow et al., 2007). Sulfate reduction (R5) causes a stronger pH increase at low pH values because of sulfate ion consumption and switches to proton production (pH decrease) at a higher pH value. In other words, the impact of sulfate reduction in sediments on pH depends on the initial conditions, that is, whether the sediment has experienced extensive denitrification and metal‐oxide reduction before initiation of sulfate reductions (Boudreau & Canfield, 1993; Meister, 2013; Soetaert et al., 2007).

## 5 Heterogeneous Buffering

While homogeneous buffering due to equilibria in solution is nearly instantaneous and can be quantified using seawater sensitivities discussed in section 3, heterogeneous buffering mechanisms involving particles occur over multiple time scales (Archer et al., 1998; Boudreau et al., 2018; Sarmiento & Gruber, 2006). For instance, proton sorption to surfaces occurs almost instantaneously, while mineral dissolution and precipitation are kinetically controlled (Lasaga, 1998). Moreover, homogeneous buffering is spatially rather uniform in the ocean because of the limited range and relative uniform distribution of salinity, dissolved inorganic carbon, and alkalinity. Heterogeneous buffering involves particles suspended in the water column, sediments deposited at the seafloor, and benthic and pelagic calcifying organisms. Calcification is dominated by pelagic organisms in the open ocean, while benthic organisms dominate in the coastal domain (Milliman, 1993; Morse et al., 2007; Morse & Mackenzie, 1990). Sediments dominate heterogeneous buffering because of the large size of this reservoir, that is, there are orders of magnitude more particles at the seafloor than suspended in the water column.

Particles suspended in the water column and deposited on the seafloor may contribute to buffering of seawater via dissolution, precipitation, and mineral surface reactions. Sorption of protons to surfaces increases alkalinity (Table 2). Reactions at the surfaces of organic, biogenic carbonate, and detrital silicate particles contribute to instantaneous buffering; alkalinity titrations of unfiltered samples containing phytoplankton and bacteria have shown that particulate matter surfaces neutralize some of the protons added (Kim et al., 2006). However, this heterogeneous buffer capacity is very limited for typical marine suspended matter concentrations. The role of surface reactions in buffering pore‐water chemistry is largely unknown although Jahnke and Jahnke (2004) identified the need to consider mineral surface reactions to properly understand sediment pH dynamics.

Dissolution, precipitation, and transformation reactions of sedimentary silicates and biogenic carbonates govern heterogeneous buffering in the ocean. Heterogeneous buffering in the ocean is dominated by carbonate compensation (Berner, 2004; Pytkowicz, 1967; Ridgwell & Zeebe, 2005), and we will focus on this as well (section 5.2), realizing that processes involving silicate minerals, such as reverse weathering and submarine weathering contribute as well, in particular on very long times scales (section 5.1; Isson & Planavsky, 2018; Mackenzie & Garrels, 1966; Sillén, 1967; Wallmann et al., 2008).

### 5.1 Silicate Reactions

The impact of seawater‐rock interactions on alkalinity is often quantified via a charge balance of major cations on the one hand and chloride, sulfate, and alkalinity on the other hand (Antonelli et al., 2017; Turchyn & DePaolo, 2019)

(27) $${\mathrm{Na}}^{+}+K^{+}+2\;{\mathrm{Mg}}^{2+}+2\;{\mathrm{Ca}}^{2+}={\mathrm{Cl}}^{-}+2\;{{\mathrm{SO}}_{4}}^{2-}+\text{alkalinity},$$

where exchanges between the univalent and divalent cations or between magnesium and calcium are considered of less importance for the balance. High‐temperature hydrothermal vents result in the removal of Ca^2+^ and SO_4_^2−^ via anhydrite precipitation and of Mg^2+^ via hydroxy‐silicate formation (Antonelli et al., 2017). The latter process generates acidity that enhances release of Ca^2+^ from basalt so that charge remains balanced. Most of the calcium released is eventually precipitated as calcium carbonate in the oceanic crust. Overall, submarine weathering results in carbon dioxide consumption and bicarbonate and calcium release (Berner, 2004; Caldeira, 1995; Staudigel et al., 1989).

Reverse weathering refers to the consumption of alkalinity and generation of protons during marine authigenic clay formation. Weathering on the continents results in the formation of cation‐depleted clay minerals which after transport and deposition at the seafloor react with major elements in seawater. Reverse weathering can be written in multiple forms, for example, kaolinite to mica transformation (Sillén, 1967),

(28) $$1.5\;{\mathrm{Al}}_{2}{\mathrm{Si}}_{2}O_{5}{\left(\mathrm{OH}\right)}_{4}\;\left(s\right)+K^{+}\Leftrightarrow {\mathrm{KAl}}_{3}{\mathrm{Si}}_{3}O_{10}{\left(\mathrm{OH}\right)}_{2}\;\left(s\right)+1.5\;H_{2}O+H^{+}$$

or cation‐poor amorphous Al‐silicate to clay (Mackenzie & Garrels, 1966),

(29) $$\text{amorphous}\;\mathrm{Al}‐\text{silicate}+{\mathrm{SiO}}_{2}\;\left(s\right)+{{\mathrm{HCO}}_{3}}^{-}+\text{cations}\Leftrightarrow \text{cation}‐\mathrm{Al}‐\text{silicate}+{\mathrm{CO}}_{2}+H_{2}O$$

The latter reaction involves the transformation of bicarbonate to carbon dioxide via reaction with cation‐depleted, acidic Al‐silicates. Reverse weathering can be written in multiple forms, but they all lower marine pH and alkalinity, and increase carbon dioxide concentrations (Isson & Planavsky, 2018).

Another submarine weathering process is induced by organic matter degradation in anoxic sediments. This degradation results in the release of carbon dioxide and dissolved organic matter (fulvic and humic acids), and these may cause dissolution of primary silicate minerals and generate high alkalinity levels (Wallmann et al., 2008). Marine weathering contributes to carbon dioxide consumption and alkalinity release and, thus, counteracts reverse weathering processes. Although seafloor weathering and in particular reverse weathering processes may have played a prominent role during past periods with warm, silica‐rich ocean waters (e.g., Precambrian), these heterogeneous buffering reactions involving silicates are considered of less importance for the present ocean than those involving carbonate minerals (Isson & Planavsky, 2018; Pytkowicz, 1967; Berner, 2004; see section 6).

### 5.2 Carbonate Compensation Dynamics

Carbonate compensation refers to the reactions between carbonate minerals and seawater, and it is instructive to distinguish between chemical and biological carbonate compensation. Chemical compensation focuses on the dissolution or preservation of carbonates at the seafloor, while biological compensation centers on the role of precipitation and its dependence on solution chemistry (Boudreau et al., 2018).

Surface oceans waters are supersaturated with respect to most carbonate minerals (Morse & Mackenzie, 1990). Supersaturation must be reduced to undersaturation, at least in the local microenvironment, before carbonate minerals will dissolve and contribute to buffering (but for surface reactions). Carbonate particles settling to the ocean floor will experience pressure increases and temperature decreases that increase solubility of carbonate minerals (Millero, 2007; Morse & Mackenzie, 1990). Moreover, subsurface ocean waters are usually richer in carbon dioxide and lower in carbonate ions because of organic matter degradation (Sarmiento & Gruber, 2006). As a consequence, seawater becomes undersaturated with respect to carbonate minerals at a certain depth and below this saturation depth mineral dissolution rates increase with depth (Ridgwell & Zeebe, 2005). At the carbonate compensation depth, the flux of carbonate particles downward is exactly balanced by the rate of carbonate dissolution (at the seafloor) with the consequence that no carbonate minerals accumulate at steady state (Boudreau, Middelburg, Hoffmann, & Meysman, 2010). The lysocline refers to the depth range between the carbonate saturation and compensation depths (Boudreau et al., 2018). Ocean buffering dynamics is reflected in changes in the depth distribution of the saturation and compensation depths (Boudreau, Middelburg, & Meysman, 2010; Ridgwell & Zeebe, 2005; Sigman et al., 1998). During periods of ocean acidification, saturation and carbonate compensation depths will shallow, causing increases in carbonate mineral dissolution and alkalinity release, counteracting the acidification. Conversely, during ocean alkalinization, saturation and compensation depths will deepen, with the result that carbonate dissolution and alkalinity release diminish (Boudreau et al., 2018; Ridgwell & Zeebe, 2005; Sigman et al., 1998).

Almost all marine carbonate minerals are of biological origin, for example, coccoliths, pteropods, and foraminifera in the open ocean and corals and mollusks in the coastal domain (Milliman, 1993; Morse & Mackenzie, 1990). Calcifying organisms consume alkalinity and any change in their activity due to alteration in environmental conditions (e.g., temperature and ocean acidification) or food web structure (e.g., food resources, predators, or viruses) consequently impacts their role in ocean buffering. This role of calcifiers in carbonate compensation has impact on buffering at multiple timescales (Boudreau et al., 2018; Caldeira & Rampino, 1993). Lower calcification rates because of ocean acidification (Gazeau et al., 2007; Kroeker et al., 2013) or global warming (Hoegh‐Guldberg et al., 2007) directly impact alkalinity removal and thus represent a rapidly operating negative feedback mechanism that will be detectable within decades (Schlunegger et al., 2019). Less calcification in the surface layer also implies less export of biogenic calcite and thus less calcite dissolution in the subsurface. Boudreau, Middelburg, Hoffmann, and Meysman (2010), Boudreau et al. (2018) provided an equation to approximate carbonate compensation depth (z_CCD_)

(30) $$z_{\mathrm{CCD}}\approx z_{\mathrm{ref}}\ln\left(\frac{F_{\mathrm{car}}\left[{\mathrm{Ca}}^{2+}\right]}{K_{\mathrm{sp}}A\beta_{\mathrm{mt}}}+\frac{\left[{\mathrm{Ca}}^{2+}\right]\;\left[CO_{3}^{2-}\right]}{K_{\mathrm{sp}}}\;\right)$$

where F_car_ is the export flux of calcium carbonate, A is the surface area of the seafloor, β_mt_ is the mass transfer of solutes across the diffusive boundary layer at the seafloor, K_sp_ is the temperature, salinity, and pressure dependent stoichiometric solubility product, [Ca^2+^] and [CO_3_^2−^] are the concentrations of dissolved calcium and carbonate ions, and z_ref_ is a scaling parameter. Lower calcification and export of calcium carbonate F_car_ will thus lead to a shallowing of the carbonate compensation depth on shorter time scales (years) but would cause additional deepening on longer timescales (10^4^ years), because of alkalinity accumulation during periods of lower calcification (Boudreau et al., 2018). This additional deepening due to biological carbonate compensation is an alternative to CO_2_‐enhanced continental weathering (Ridgwell, 2007; Zeebe et al., 2009) for CaCO_3_ overshooting in the geological record (Luo et al., 2016).

## 6 Processes Governing Alkalinity in the Ocean

The distribution of alkalinity often covaries with salinity. This is logical because oceanographic processes impacting salinity by freshwater addition (such as precipitation, river, and groundwater discharge and ice melting) or removal (e.g., evaporation and ice formation) also impact alkalinity. These processes are most intense in surface waters that are exposed to the atmosphere, cryosphere, and riverine inputs. It is for this reason that alkalinity can be predicted quite well in ocean surface water using salinity and temperature (Lee et al., 2006; Millero et al., 1998). Alkalinity is often normalized to salinity to infer the other processes affecting alkalinity such as calcite production and dissolution (Carter et al., 2014; Feely et al., 2004; Sarmiento & Gruber, 2006). However, salinity normalization might induce biases, because of regional differences in salinity‐alkalinity relationships (Friis et al., 2003; Jiang et al., 2014).

**Figure 6:**
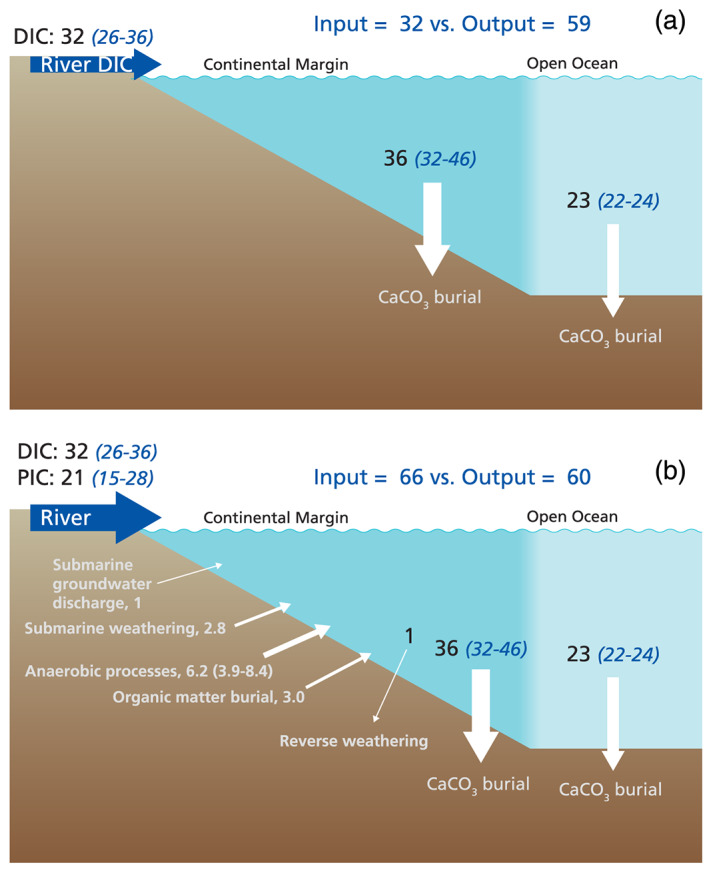
Alkalinity balance of the ocean (fluxes are in Tmol year^−1^). (a) Traditional alkalinity balance considering river input of DIC and calcium carbonate burial. (b) Revised oceanic alkalinity balance considering additional sources and sinks based on Table 3.

Whole ocean alkalinity is largely governed by the balance between ions generated by weathering and removal of ions via the formation of calcium carbonate. Continental weathering on land generates cations that are charge balanced by alkalinity (Berner & Berner, 2012; Mackenzie & Garrels, 1966; Turchyn & DePaolo, 2019). Rivers and groundwater deliver cations and alkalinity to the ocean where calcifiers produce skeletons and remove alkalinity. On million‐year timescales, this is usually represented by the Urey‐Ebelman reaction (Berner, 2004; Urey, 1952)

(31) $${\mathrm{CO}}_{2}+{\text{CaSiO}}_{3}\to {\text{CaCO}}_{3}+{\mathrm{SiO}}_{2,}$$

which illustrates the net transfer of carbon from the atmosphere to the sedimentary record. Accordingly, over geological timescales, one would expect that riverine delivery of alkalinity to the ocean is balanced by burial of carbonate in marine sediments (Figure 6a).

Estimates of riverine alkalinity delivery range from 26 to 36 Tmol year^−1^ (supporting information S3 and Table 3) and have been derived either from river DIC export or global estimates of CO_2_ consumption by chemical weathering of silicate and carbonate rocks (Berner et al., 1983; Gaillardet et al., 1999; Hartmann et al., 2014; Ludwig et al., 1996; Li et al., 2017; Meybeck, 1987; 1998; Suchet et al., 2003). River biogeochemists normally assume that bicarbonate equals the alkalinity and dissolved inorganic carbon (Raymond & Hamilton, 2018; Suchet et al., 2003). Consequently, global estimates of DIC delivery to the ocean can be used as a proxy for alkalinity transfer from weathering to the ocean. Carbonate burial estimates range from 18 to 34 Tmol C year^−1^ (supporting information S3), with clear consensus about carbonate burial in the deep sea of 11–12 Tmol C year^−1^, while ocean margin contributions vary from 6 to 23 Tmol C year^−1^ (Iglesias‐Rodriguez et al., 2002; Milliman, 1993; Milliman & Droxler, 1996; Morse & Mackenzie, 1990; O'Mara & Dunne, 2019; Smith, 2013; Smith & Mackenzie, 2016; Wollast, 1994). Calcium carbonate formation involves the consumption of two moles of alkalinity per mole of carbon (Table 2), indicating an imbalance between alkalinity inputs from chemical weathering (26–36 Tmol year^−1^) and alkalinity outputs by carbonate burial (54–62 Tmol year^−1^), the latter based on carbonate (carbon) burial equal to 27–31 Tmol C year^−1^ (Figure 6 and supporting information S3). This imbalance has been identified before based on Ca and HCO_3_^−^ budgets of the ocean (Berner & Berner, 2012).

**Table 3:** Global Alkalinity Balance of the Ocean (Tmol year^−1^)

Alkalinity sources/sinks	Riverine DIC and carbonate burial balance	Complete alkalinity balance
Riverine DIC	32	32
Riverine PIC		21
Submarine Groundwater		1
Submarine Silicate		2.8
Sulfur Burial		4.7
Denitrification		1.5
Organic Matter Burial		3
Total Sources	32	66
Open Ocean Carbonate Burial	23	23
Ocean Margin Carbonate Burial	36	36
Reverse Weathering		1
Total Sinks	59	60
Imbalance	27	−6

*Note*. Supporting Information S3 provides a detailed documentation for the various terms, including the range.

This imbalance of 18 to 36 Tmol year^−1^ can be explained in three ways. One, the present‐day ocean may not be at steady state regarding alkalinity (Milliman, 1993). The inventory of alkalinity in the ocean is about 3.15 10^18^ mol, based on a total ocean volume of 1.34 10^18^ m^3^ and a mean ocean alkalinity of about 2.35 mol m^−3^ (Sarmiento & Gruber, 2006). Accordingly, the residence time of alkalinity is about 88–121 ky with respect to a riverine input of 26–36 Tmol year^−1^. Consequently, the modern ocean alkalinity budget may still be recovering from last glacial sea level drop and related shifts in carbonate burial/exposure on continental shelves. Two, carbonate burial in ocean margin sediments may be lower than consensus values (16–20 Tmol C year^−1^; Milliman & Droxler, 1996; Iglesias‐Rodriguez et al., 2002; Smith, 2013; Smith and Mackenzie, 2016; O'Mara & Dunne, 2019). Morse and Mackenzie (1990) (their Figure 5.1) reported a long‐term carbonate burial of about 6 Tmol C year^−1^ in ocean margin sediments. Total alkalinity removal by carbonate burial would then be about 34 Tmol year^−1^ (2*(6 + 11)) and result in a balanced budget. Van der Ploeg et al. (2019) reported an alkalinity removal via Cenozoic marginal carbonate burial of 14.3 Tmol year^−1^ by balancing riverine and anaerobic mineralization inputs with marginal and deep‐sea carbonate burial. However, there is consensus that modern carbonate burial in ocean margins is about 16–20 Tmol C year^−1^ (supporting information S3), hence an alkalinity removal of 32–40 Tmol year^−1^. Three, this simple depiction of riverine alkalinity balancing calcium carbonate burial in marine sediment (Figure 6a) requires revision because of additional alkalinity inputs from land (e.g., riverine particulate inorganic carbon and groundwater alkalinity) or from marine sediments (Figure 6b).

Rivers deliver elements to the ocean not only in dissolved form but also in particulate form (Martin & Meybeck, 1979). If riverine particulate carbonates are unreactive, they will eventually be buried in marine sediments and are then implicitly included in the marine carbonate burial term. Similarly, if riverine particulate carbonates are reactive and dissolve, they represent an alkalinity source and should be added to the input. Irrespective of their fate, transfer of particulate inorganic carbon from rivers to the ocean would reduce the imbalance. There are very few data on the global particulate inorganic carbon input to the ocean. On the basis of a very small data set, Meybeck (1987) reported a PIC flux of 14.2 Tmol C year^−1^ and this is still used in global carbon assessments (Li et al., 2017). This PIC flux implies an additional alkalinity source of 28.4 Tmol year^−1^. This is not only of similar magnitude as the alkalinity delivered in dissolved form, but it would also close the modern ocean alkalinity budget (Table 3). This large, so far overlooked, alkalinity input to the ocean is however poorly constrained. Using the global sediment delivery estimate (19 Pg year^−1^; range 11–27 Pg year^−1^) of Beusen et al. (2005) and the average of PIC content of US rivers (0.47 wt% C) from Canfield (1997), a lower riverine PIC delivery is estimated (7.4 Tmol C year^−1^; range 4.3–10.6 Tmol C year^−1^). However, this would still correspond to an alkalinity flux of almost 15 Tmol year^−1^. This difference is primarily due to difference in PIC content of suspended particles (0.9 wt% for Meybeck (1982) vs. 0.47 wt% in US river data base; Canfield, 1997). The average of these two uncertain numbers is used for the alkalinity budget (Figure 6b and Table 3).

Part of the freshwater return flow to the ocean occurs via groundwater discharge rather than via rivers (Slomp & Van Cappellen, 2004), and this is an additional alkalinity input. Zhou et al. (2019) reported a global freshwater submarine discharge flux of 489 km^3^ year^−1^, which corresponds to about 1.3% of the global river discharge and is significantly lower than the 5% adopted by Slomp and van Cappellen (2004). Combining these fractions with assumptions about the alkalinity of groundwater, one to three times that of river water (Zhang & Planavsky, 2019), we estimate a global fresh groundwater alkalinity input of about 1 Tmol year^−1^, range 0.4–4.7 Tmol year^−1^ (supporting information S3).

Besides these additional alkalinity inputs from riverine PIC and submarine groundwater discharge, there is also alkalinity production and consumption in marine sediments due to anaerobic degradation of organic matter, organic matter burial, and reactions involving silicates (Ben‐Yaakov, 1973; Berner et al., 1970; Boudreau & Canfield, 1993; Hu & Cai, 2011; Soetaert et al., 2007; Wallmann et al., 2008). Although we understand the impact of individual processes on TA quite well (see sections 4 and 5), the overall effect of these processes on ocean TA is more difficult to assess because of the tight coupling between alkalinity generating and consuming processes within a sediment column. For instance, dissimilatory sulfate reduction and sulfate reduction coupled to anaerobic methane oxidation generate TA (Table 2), but most of the sulfide and ammonium generated during these processes are reoxidized, resulting in alkalinity consumption (Table 2). Consequently, net overall impact of sedimentary sulfate reduction on ocean alkalinity is limited to the small fraction of reduced sulfur that is eventually buried (Gustafsson et al., 2019; Hu & Cai, 2011; Krumins et al., 2013; Wallmann et al., 2008). Moreover, alkalinity released from the sediment in the form of reduced substances (e.g., ammonium and sulfide) that are subsequently oxidized (e.g., nitrification and sulfide oxidation) in the water column does not contribute to the whole ocean alkalinity balance either (Hu & Cai, 2011).

In the context of the global ocean alkalinity balance, anaerobic alkalinity production can conceptually be considered as an anion charge transfer process, as discussed in section 4 (Ben‐Yaakov, 1973; Hu & Cai, 2011). During denitrification (reduction of nitrate to dinitrogen gas), the charge of nitrate is transferred to bicarbonate and thus increases alkalinity. Similarly, the reduced sulfur buried in marine sediments initially entered the ocean as a sulfate ion; this implies a charge transfer to bicarbonate. In contrast, solid phase oxidants such as iron and manganese oxides enter the ocean uncharged and leave the ocean in an uncharged solid form (e.g., FeS_2_), with no impact on global ocean alkalinity. Accordingly, it is only the charge transfer from land‐derived nitrate and sulfate to bicarbonate produced by anaerobic respiration that matters for the whole ocean alkalinity balance (Hu & Cai, 2011).

Net alkalinity production due to denitrification/anammox is thus about 1.5 Tmol year^−1^ (Hu & Cai, 2011) based on a river nitrate input of 21 Tg N year^−1^ (Seitzinger et al., 2006). The net alkalinity production due to sulfate reduction is derived from the burial of sulfur in marine sediments because most of the sulfate produced is reoxidized (Jørgensen, 1977, 1982). Estimates of sulfur burial are based on organic carbon burial and vary from 1.2 (Berner, 1982) to 3.4 Tmol S year^−1^ (supporting information S3), implying a potential alkalinity source of 2.4 to 6.9 Tmol year^−1^ to the ocean. These are upper estimates because part of the net alkalinity generated within sediments results in authigenic carbonate formation and does not contribute to ocean alkalinity. Accordingly, the total contribution of anaerobic biogeochemical processes to the ocean alkalinity balance varies between 3.9 and 8.4 Tmol year^−1^.

Primary production based on new nitrogen (e.g., nitrate) is an alkalinity source, while aerobic respiration accompanied by nitrification represent an alkalinity sink (Table 2). If all organic matter produced were respired there would be no impact on alkalinity, but a small part of the organic matter produced in the photic zone is eventually buried in marine sediments. Based on organic carbon burial estimates of Berner (1982) and Burdige (2007), we estimate a net alkalinity production of 3 Tmol year^−1^ (see supporting information S3).

Quantifying the contribution of sediment silicate reactions to ocean alkalinity is also complicated by multiple alkalinity production and consumption processes: reverse weathering in surface sediments, marine weathering at depth, in particular in the methanogenic zone (Wallmann et al., 2008), and ocean crust weathering (Berner, 2004; Caldeira, 1995; Staudigel et al., 1989). Moreover, the alkalinity generated at depth by mineral weathering results in authigenic carbonate formation. Although alteration of oceanic crust is likely on the order of 2 Tmol year^−1^, most bicarbonate generated is precipitated as calcite and does not contribute to ocean alkalinity. Reverse weathering is a sink of alkalinity (e.g., Equations . 28 and 29) on the order of about 0.5–1.5 Tmol year^−1^ (Isson & Planavsky, 2018) but varies locally depending on the supply of materials (Michalopoulos & Aller, 1995, 2004; Rahman et al., 2016). Wallmann et al. (2008) reported high rates of submarine weathering of 3.3 to 13.3 Tmol year^−1^. Their estimate is based on the assumption that all carbon dioxide produced during methanogenesis is converted to bicarbonate and that one third is removed by authigenic carbonate formation and two third is released as alkalinity to the ocean. These numbers are likely too high given that these are based on global methane production rates of 5 (Reeburgh et al., 1993) and 20 Tmol C year^−1^ (Hinrichs & Boetius, 2002). More recent estimates for marine methane formation are 2.8 Tmol year^−1^ (Egger et al., 2018) and 0.3 to 2.1 Tmol C year^−1^ (Wallmann et al., 2012). This would lower submarine weathering alkalinity input to 2–3 Tmol year^−1^ (supporting information S3).

Figure 6a and Table 3 clearly show that the ocean alkalinity budget based on the balance between riverine alkalinity inputs and carbonate burial at the ocean floor is imbalanced by about 27 Tmol year^−1^ and provides only part of the story. Additional alkalinity from riverine PIC delivery (about 21 Tmol year^−1^) and anaerobic mineralization (about 6.2 Tmol year^−1^) provides the majority of the alkalinity to balance the budget with minor additional inputs from submarine groundwater discharge (about 1 Tmol year^−1^), organic matter burial (about 3 Tmol year^−1^), and silicate interactions (about 1 Tmol year^−1^; submarine weathering minus reversed weathering).

## 7 Conclusions

Alkalinity is a central concept in ocean buffering and it is of the utmost importance to understand and quantify its role in carbon dioxide uptake, carbonate mineral formation, and ocean buffering during times of global change. In section 2, we have shown that it is instructive to distinguish between measurable titration alkalinity (TA) that is based on a proton balance and CBA. This distinction is needed to understand and quantify the impact of biogeochemical processes such as calcification or primary production on alkalinity.

Although much progress has been made in accurate and reproducible measurements of titration alkalinity, quantification of ocean buffering through the use of buffer or sensitivity factors is underexplored, except for the Revelle sensitivity factor (Sundquist et al., 1979) and the acid–base buffer capacity (Weber & Stumm, 1963). This is unfortunate because such a sensitivity analysis is critical to attribute changes in pCO_2_ and/or pH to physical (temperature and salinity) and chemical/biological changes (alkalinity and dissolved inorganic carbon). In section 3, we have provided a systematic treatment of these sensitivity factors and identified and resolved inconsistent terminology. Together with the availability of public domain packages such as *seacarb* (Gattuso et al., 2019) and *AquaEnv* (Hofmann, Soetaert, et al. (2010b)) that facilitate their calculation, this provides researchers with the tools to understand and predict changes in ocean chemistry. This can be retrodiction of past or prediction of future pH changes due to ocean acidification or prediction of future pH, pCO_2_, or carbonate saturation values upon alkalinity manipulation during geo‐engineering (Renforth & Henderson, 2017).

While these sensitivity factors provide a powerful approach to deal with equilibrium reactions, they are less useful when buffering is provided by interactions with slowly reacting solids or by changes in biological processes (e.g., calcification). These heterogeneous buffering reactions are dominated by calcium carbonate formation and dissolution and normally discussed in terms of ocean carbonate compensation dynamics (Boudreau et al., 2018). Traditionally, the focus has been on the role of carbonate mineral dissolution in the water column and sediments, that is, chemical dissolution, which provides long‐term buffering (Archer et al., 1998, 2009; Ridgwell & Zeebe, 2005; Sigman et al., 1998). However, ocean acidification or warming also impact calcification (e.g., coral bleaching). This biological carbonate compensation mechanism has implications on the short term (months) for the carbonate compensation depth and on the longer term (kyr) for alkalinity accumulation in the ocean (Boudreau et al., 2018). Homogeneous buffering, chemical and biological carbonate compensation, and weathering feedbacks together govern the long‐term fate of anthropogenic carbon dioxide (Archer et al., 1998, 2009; Boudreau, Middelburg, Hoffmann, & Meysman, 2010). The very same processes have also been involved in the recovery of ocean chemistry to carbon perturbations in the past (Boudreau et al., 2018; Ridgwell & Zeebe, 2005).

Ocean alkalinity is controlled by multiple processes operating over multiple time scales complicating the elucidation of the present‐day budget (Table 3) as well as reconstructions of alkalinity, carbon, and pH of the past ocean. Nevertheless, some studies (Boudreau et al., 2019; Caves et al., 2016; Tyrrell & Zeebe, 2004; Zeebe & Tyrrell, 2019) have reported alkalinity reconstructions for the last 50–60 million years indicating relatively minor fluctuations, for example, between 1.5 and 3 mM. The Urey‐Ebelmen concept implies that riverine alkalinity delivery should be balanced by carbonate burial in the ocean on geological timescales (Figure 6a). The imbalance of the present‐day ocean alkalinity budget between riverine DIC input and marine carbonate burial cannot be resolved by including alkalinity delivery by submarine groundwater or within ocean alkalinity production by anaerobic mineralization or submarine weathering. However, the alkalinity budget can readily be balanced by including alkalinity input to the ocean through riverine particulate inorganic carbon delivery (Table 3 and Figure 6b). The global riverine flux of PIC is poorly known, but reasonable estimates (7.5 to 15 Tmol C year^−1^; corresponding to an alkalinity flux of 15–30 Tmol year^−1^) indicate that it is similarly sized as the global riverine DIC flux (26–36 Tmol year^−1^). This not only urges detailed research to better constrain this flux but also implies a reconsideration of past and present ocean alkalinity budgets that ignore physical weathering products delivered to the ocean.

### Glossary

- *Acid*: substance donating a proton
- *Base*: substance accepting a proton
- *Conjugated acids/bases*: substances that only differ by one proton
- *strong acid*: acid that donates all protons in natural water
- *weak acid*: acid that partly dissociates to conjugate base and proton in water
- *pH:* ‐logarithm_10_ of proton concentration (H^+^)
- *pK*: ‐logarithm_10_ of equilibrium constant (K)
- *Bjerrum plot*: graph showing the distribution of acids and their conjugated bases as a function of pH
- *alkalinity*: the excess of proton acceptors (over proton donors) in a solution
- *titration alkalinity (TA)*: measurable alkalinity based on a proton balance
- *charge balance alkalinity (CBA)*: alkalinity based on excess of proton exchanging anions over cations, also known as excess negative charge (ENC)
- *buffer*: solution with a mixture of weak acids and conjugated bases that resist changes by transferring protons
- *homogeneous buffer*: solution resisting drastic changes by rearrangement of protons in solution phase only
- *heterogeneous buffer:* a buffer system comprising both a solution and particles
- *buffer capacity*: ability of a solution to resist changes, also known as buffer index or intensity.
- *sensitivity factor*: change in output due to change in input, also known as chemical buffer factor, and the inverse of buffer capacity
- *carbonate compensation*: response of carbonate production and dissolution processes in the ocean upon changes
- *proton balance*: mass balance of protons
- *proton acceptor level*: the number of protons that can be accepted for an acid–base system at a specific pH, the reverse is known as the proton level
- *reference level species*: the major species of an acid–base system present at the reference level (e.g., pH = 4.5)
- *Revelle factor*: a sensitivity factor expressing the change in carbon dioxide to the change in dissolved inorganic carbon

## Author Contributions

J. J. M. designed the research and wrote the manuscript with contributions from K. S. and M. H., in particular for sections 2, 4. K. S. and M. H. performed the calculations presented in Figures 1, 2, 3, 4, 5.

## Supporting information

Supporting Information S1

Software S1
