# Supplementary material for: Ocean Alkalinity, Buffering and Biogeochemical Processes
Source: Rev Geophys. 2020 Sep;58(3):e2019RG000681. doi: 10.1029/2019RG000681 (PMC7391262; doi:10.1029/2019RG000681)
Supplement: Supplementary file 1 — Supporting Information S1 [file ROG-58-e2019RG000681-s001.pdf]

Reviews of Geophysics

Supporting Information for

Ocean Alkalinity, Buffering and Biogeochemical Processes

Jack J. Middelburg[a], Karline Soetaert[b] and Mathilde Hagens[c]

a. Department of Earth Sciences, Geosciences, Utrecht University, the Netherlands
b. Department of Estuarine and Delta Systems, Royal Netherlands Institute for Sea Research (NIOZ Yerseke) and Utrecht University, Yerseke, The Netherlands
c. Soil Chemistry and Chemical Soil Quality, Wageningen University, the Netherlands

Contents of this file

Text S1: Solving carbonate equilibria via proton and charge balances
Text S2: Relations between various sensitivity factors reported in the literature
Text S3: Alkalinity balance of the ocean

Additional Supporting Information (Files uploaded separately)

R script used for calculations and generation of Figures 1 to 5

Introduction

The supporting information contains three sections. The first section (S1) elaborates the differences between proton and charge balances to solve carbonate equilibria and complements section 2. The second section (S2) presents explicit links between sensitivity and buffer factors reported in the literature and provides the basis for Table 1. The third section (S3) provides details on alkalinity sources and sinks in the ocean.

Text S1: Solving carbonate equilibria via proton and charge balances.

Solving ionic equilibrium problems implies balancing the number of species in solution with the number of equilibrium relations, mass and charge balances (Butler, 1964). The two alkalinity entities (titration alkalinity and charge balance alkalinity) are rooted in the use of either a proton mass balance or charge balance to obtain the needed number of equations. Consider pure water in which the water is dissociated into protons and hydroxide ions:

$H_2O \Leftrightarrow H^+ + OH^-$ (eq. 1.1)

This reaction occurs virtually immediately and one can thus assume equilibrium between the three species (H_2O, H^+, OH^-):

$$K'_w = \frac{H^+ \cdot OH^-}{H_2O} \quad \text{(eq. 1.2a)}$$

where K'_w, the equilibrium constant for water self-ionisation, governs the distribution between protons and hydroxide ions. Water as the liquid medium is always present with a constant concentration of ~55.4 M (998 gr H_2O L^{-1}/18 gr H_2O mol^{-1}) and implicitly included in the equilibria. Accordingly, eq. 2a becomes

$$K_w = H^+ \cdot OH^- \quad \text{(eq. 1.2b)},$$

where K_w= 55.4* K'_w= 10^{-14}, ignoring activity coefficients. Besides eq. 1.2b we need one additional equation to obtain the concentration of two species (H^+ and OH^-). There are two alternatives. The first option is the balance between positive and negative ions because water is electrically neutral:

$H^+ = OH^-$ (eq.1.3).

Alternatively, the proton condition, i.e. a proton mass balance, can be used. Self-ionisation of water results in the formation of one proton and one hydroxide ion, hence eq. 1.3 is again obtained. The proton condition and charge balance are identical for this trivial case for pure water with pH=7.

Next, we consider pure water to which a known amount of carbonic acid (H_2CO_3) has been added. Carbonic acid is a weak diprotic acid and partly dissociates first into a bicarbonate ion (HCO_3^-) and a proton and subsequently the bicarbonate is dissociated partly into a carbonate ion (CO_3^{2-}) and a proton. The relevant reactions are:

$H_2CO_3 \Leftrightarrow HCO_3^- + H^+$ (eq. 1.4)

$HCO_3^- \Leftrightarrow CO_3^{2-} + H^+$ (eq. 1.5)

for which we can write equilibrium relations:

$$K_1 = \frac{HCO_3^- \cdot H^+}{H_2CO_3} \quad \text{(eq. 1.6)}$$

and $K_2 = \frac{CO_3^{2-} \cdot H^+}{HCO_3^-}$ (eq. 1.7),

where K_1 and K_2 are the first and second stoichiometric equilibrium constants ($10^{-6.35}$ and $10^{-10.3}$).

Accordingly, for the CO_2-H_2O system we have five unknown species (H_2CO_3, HCO_3^-, CO_3^{2-}, OH^- and H^+) and three equilibrium relations: water self-ionisation (eq.1.2b), and the first and second equilibria of carbonic acid dissociation (eq. 1.6, 1.7). Moreover, we know the total mass of carbonic acid added ($\Sigma CO_2 = H_2CO_3 + HCO_3^- + CO_3^{2-}$). To solve the system, we need one additional relation and again two alternative routes can be followed. The first option balances the positive charge of protons with the negative charge of hydroxide, bicarbonate and carbonate ions.

$H^+ = OH^- + HCO_3^- + 2CO_3^{2-}$ (eq.1.8).

Note that the carbonate ion is counted twice in the charge balance because of its double charge. Alternatively, the proton condition can be used because protons are involved in all three reactions (eq. 1.1, 1.4, 1.5) and their total mass is conserved. The proton condition is given by the sum of the protons released when water and carbonic acid dissociate to their equilibrium distribution (Butler, 1964, 1982):

$H^+ = H^+_{H2O} + H^+_{H2CO3}$ (eq.1.9a).

or its equivalent $H^+ = OH^- + HCO_3^- + 2\ CO_3^{2-}$ (eq.1.9b).

This equation is called a proton condition because all species on the left-hand side have excess protons relative to the (reference) species of the recipe (H_2O and H_2CO_3), while species on the right-hand side are deficient in protons. The species H_2O and H_2CO_3 are the zero level of protons for this system and each species is multiplied with the number of protons needed to convert them to the zero-proton level. The proton condition is thus similar to the charge balance, the difference being that excess/deficiency of protons rather than electrons are counted. The proton condition is usually presented as the total proton concentration (TOTH; Morel and Hering, 1993):

$TOTH = H^+ - OH^- - HCO_3^- - 2\ CO_3^{2-}$ (eq. 1.10).

Independent whether the charge balance, proton condition or total proton concentration equation is used, the system is now fully defined with 5 unknown species linked via 5 equations.

Adding NaCl to this solution will not only increase the number of unknown species from 5 to 7, but also adds to two conservation equations, one for total Na^+ and one for total Cl^-. Dissolution of NaCl does not impact the proton mass balance (eq. 1.9, 1.10), because Na^+ and Cl^- are not involved in proton exchange. However, it does imply a revision of the charge balance (eq. 1.8) to:

$Na^+ + H^+ = OH^- + HCO_3^- + 2CO_3^{2-} + Cl^-$ (eq. 1.11).

Rearranging this charge balance for the system H_2O-H_2CO_3-NaCl to obtain the ions Na^+ and Cl^- on the left-hand side, because they are invariant to changes in pH, temperature and pressure (i.e. conservative), yields the negative of TOTH on the right-hand side:

$CB = Na^+ - Cl^- = OH^- + HCO_3^- + 2CO_3^{2-} - H^+ = -TOH$ (eq. 1.12).

This equation links –TOH, the definition of titration alkalinity (Dickson, 1981; Morel and Hering, 1993), with the charge balance of conservative ions (CB).

Proton mass balances are always relative to a proton reference level. Equations (1.9 and 1.10) are relative to H_2CO_3 (because H_2CO_3 has been added) and in this case, the proton condition is identical to the charge balance. However, if we had added $NaHCO_3$ and HCO_3^- were the reference level, the proton balance would be:

$TOTH = H^+ + H_2CO_3 - OH^- - CO_3^{2-}$ (eq. 1.13),

and the charge balance and proton balance would differ by the total concentration of carbonic acid ((ΣCO_2). Adding additional substances to our mixture to produce seawater will increase the number of species, equilibria among the species and mass conservation equations, but there is always the need for either a charge balance or proton condition to close the system. The (seawater) titration alkalinity definitions of Dickson (1984) and TOTH of Morel and Hering (1993) are based on the proton condition, while the explicit conservative expression of total alkalinity (Zeebe and Wolf-Gladrow, 2001; Wolf-Gladrow et al., 2007) and the excess negative charge (Soetaert et al., 2007) are based on charge balance equations.

Text S2 Relations between various sensitivity factors reported in the literature

In this section, we present the relations between sensitivity factors reported in the literature and that are listed in Table 1.

Relations among the various sensitivity factors reported (as buffer factors) by Frankignoulle (1994).

Hagens and Middelburg (2016a) derived from Frankignoulle's (1994) work that

$$\frac{\partial H^+}{\partial HCO_3^-} = \frac{\partial H^+}{\partial DIC} + \frac{\partial H^+}{\partial TA}$$

and

$$\frac{\partial H^+}{\partial CO_3^{2-}} = \frac{\partial H^+}{\partial DIC} + 2\frac{\partial H^+}{\partial TA}$$

We can translate this to pH knowing that

$$\frac{\partial H^+}{\partial X} = \frac{\partial H^+}{\partial pH}\frac{\partial pH}{\partial X}$$

which results in:

$$\frac{\partial pH}{\partial HCO_3^-} = \frac{\partial pH}{\partial DIC} + \frac{\partial pH}{\partial Alk} \qquad (\Phi_B = \Phi + \Phi_H)$$

and

$$\frac{\partial pH}{\partial CO_3^{2-}} = \frac{\partial pH}{\partial DIC} + 2\frac{\partial pH}{\partial Alk} \qquad (\Phi_C = \Phi + 2\Phi_H)$$

where the symbols are from Frankignoulle (1994).

Similarly, for pCO_2:

$$\frac{\partial pCO_2}{\partial HCO_3^-} = \frac{\partial pCO_2}{\partial DIC} + \frac{\partial pCO_2}{\partial Alk} \qquad (\Pi_B = \Pi - \Pi_H)$$

and

$$\frac{\partial pCO_2}{\partial CO_3^{2-}} = \frac{\partial pCO_2}{\partial DIC} - 2\frac{\partial pCO_2}{\partial Alk} \qquad (\Pi_C = \Pi - 2\,\Pi_H)$$

But also the sensitivity factors with respect to the carbonate species are related:

$$\frac{\partial pH}{\partial HCO_3^-} = 0.5\left(\frac{\partial pH}{\partial CO_2} + \frac{\partial pH}{\partial CO_3^{2-}}\right) \qquad \left(\Phi_B = 0.5(\Phi_D + \Phi_C)\right)$$

$$\frac{\partial pCO_2}{\partial HCO_3^-} = 0.5\left(\frac{\partial pCO_2}{\partial CO_2} + \frac{\partial pCO_2}{\partial CO_3^{2-}}\right) \qquad \left(\Pi_B = 0.5(\Pi_D + \Pi_C)\right)$$

$$\frac{\partial lnpCO_2}{\partial lnHCO_3^-} = 0.5\left(\frac{\partial lnpCO_2}{\partial lnCO_2} + \frac{\partial lnpCO_2}{\partial lnCO_3^{2-}}\right) \qquad \left(\beta_B = 0.5(\beta_D + \beta_C)\right)$$

Relations between factors of Egleston et al. (2010) and Frankignoulle (1994)

Using their symbols, Frankignoulle's factors on the left-hand side are related to those of Egleston et al. (2010) on the right-hand side:

$$\beta_D = \frac{DIC}{\gamma_{DIC}}$$

$$\Pi_H = \frac{-pCO_2}{\gamma_{Alk}} = \frac{-CO_2}{K_0\gamma_{Alk}}$$

$$\Pi_D = \frac{pCO_2}{\gamma_{DIC}} = \frac{CO_2}{K_0\gamma_{DIC}}$$

$$\Phi_H = \frac{1}{\ln{(10)}\beta_{Alk}}$$

$$\Phi_D = \frac{-1}{\ln{(10)}\beta_{DIC}}$$

$$\beta_C = \frac{DIC}{\omega_{DIC}}$$

$$\Pi_C = \frac{pCO_2}{\omega_{DIC}} = \frac{CO_2}{K_0\omega_{DIC}}$$

Relations between factors of Sarmiento and Gruber (2006), Frankignoulle (1994) and Egleston et al. (2010)

The factor β_D of Frankignoulle is identical to γ_{DIC} of Sarmiento and Gruber:

$$\beta_D = \gamma_{DIC} = \frac{DIC}{pCO_2}\frac{\partial pCO_2}{\partial DIC}$$

but differs from the similarly named γ_{DIC} of Egleston et al.:

$$\beta_D = \frac{DIC}{\gamma_{DIC}}$$

Another inconsistency relates to γ_{TA} of Sarmiento and Gruber:

$$\gamma_{TA} = \frac{TA}{pCO_2}\frac{\partial pCO_2}{\partial TA}$$

which again differs from γ_{TA} of Egleston et al.:

$$\gamma_{TA} = \left(\frac{\partial TA}{\partial lnCO_2}\right)$$

Relations between isocapnic quotient (Q) of Humphreys et al. (2018) and general sensitivity theory of Hagens and Middelburg (2016a)

Recently, Humphreys et al (2018) introduced another sensitivity factor, the isocapnic quotient (Q) defined as:

$$Q = \frac{\partial TA}{\partial DIC}$$

This isocapnic quotient is fully consistent with the general sensitivity approach of Hagens & Middelburg (2016a). Starting from their table 3:

$$\frac{\partial TA}{\partial X} = \left(\frac{\partial X}{\partial TA}\right)^{-1} = \frac{A_X^2 + \left(H^+\left(\frac{\partial TA}{\partial H^+}\right)_X - nA_X\right)TotX}{-X(-A_X + nTotX)}$$

Here, TotX refers to the total concentration of the acid-base system of interest, X to the species of interest of that acid-base system (which equals the reference species for A_T (X_{ref}) in the case a change in TotX is specified), n to the stoichiometric factor in the contribution of X to A_T (which equals 0 in the case a change in TotX or X_{ref} is specified) and A_X to the contribution of all species of TotX to A_T.

For this specific case with DIC as state variable (i.e., X = CO_2 and n = 0) and total borate concentration (TotB) as reaction invariant contributing to TA, this translates into:

$$\frac{\partial TA}{\partial DIC} = \frac{A_C^2 + H^+\left(\frac{\partial TA}{\partial H^+}\right)_X DIC}{[CO_2]A_C}$$

With

$$\left(\frac{\partial TA}{\partial H^+}\right)_X = \frac{-1}{H^+}\left(HCO_3^- + 4CO_3^{2-} + B(OH)_4^- \frac{B(OH)_3}{TotB}\right)$$

Which is fully equivalent to Eq. 8 of Humphreys et al (2018):

$$\frac{\partial TA}{\partial DIC} = \frac{\left(K_1H^+DIC + 4K_1K_2DIC + K_wH^+ + H^{+^3}\right)(K_B+H^+)^2 + K_BTotBH^{+^3}}{K_1DIC\ (H^+ + 2K_2)(K_B+H^+)^2}$$

Section S3 Alkalinity balance of the ocean.

Table 3 presents a concise, consensus budget for ocean alkalinity. Some of the individual terms have a range and others have been calculated in this study. This supplementary section provides an overview and rationale for most terms.

3.1 Alkalinity input to the ocean

Riverine alkalinity supply

Estimates of riverine alkalinity supply are normally assumed to be identical to riverine DIC supply to the ocean because DIC≈TA at river pH values. Riverine DIC transport to the ocean is rather well constrained as published numbers vary from 26.6 to 36.3 Tmol y^{-1}: 32 Tmol y^{-1} (Meybeck, 1987); 26.6 Tmol y^{-1} (Ludwig et al., 1996); 27.4 Tmol y^{-1} (Ludwig et al., 1998); 36.3 Tmol y^{-1} (Gaillardet et al., 1999); 30.1 Tmol y^{-1} (Suchet et al., 2003); 33.8 Tmol y^{-1} (Hartmann et al., 2014) and 34.2 Tmol y^{-1} (Li et al., 2017). Some of this consistency may be simply due to the use of the same data as basis for extrapolation or calibration of the model, but various approaches have been used to obtain the final global numbers (spatially resolved or not, data driven vs. model). The average river TA flux is 32 Tmol y^{-1} and used in Table 3.

Submarine groundwater supply

Submarine groundwater supply of alkalinity to the ocean is poorly constrained. Combining the recent Zhou et al. (2019) estimate for global freshwater submarine discharge of 489 km^3 y^{-1}, i.e. ~1.3% of global river discharge of 37,288 km^3 y^{-1} (Berner and Berner, 2012), with the average river TA of ~ 0.85 mM (31.5 Tmol/37288 km^3), we estimate a TA flux of 0.4 Tmol y^{-1}. However, groundwaters usually have higher TA levels because of carbonate dissolution and anaerobic processes. Considering that groundwater TA is three times that of rivers (Zhang and Planavasky, 2019), we estimate a submarine groundwater supply of 1.2 Tmol y^{-1}. Recently, Zhang and Planavasky (2019) reported a much higher contribution ranging from 7.4 to 83 Tmol y^{-1}. This difference is primarily due to uncertainty in submarine groundwater discharge estimates. Our conservative estimate is based on the Zhou et al. (2019) estimate of global freshwater submarine discharge, which is lower than the often used 5% of global river discharge estimate of Slomp and van Cappellen (2005). Combining this higher discharge rate with average river TA, we obtain 1.6 Tmol y^{-1}. Accordingly, the global submarine groundwater supply of alkalinity to the ocean adopted for Table 3 is about 1 Tmol y^{-1}.

Submarine weathering

Weathering of silicates in the ocean represents a sink of carbon dioxide and a source of alkalinity. Ocean crust weathering acts a sink of carbon dioxide, but most of the alkalinity generated is removed via the precipitation of calcium carbonate (Caldeira, 1995; Berner, 2004). Submarine weathering of continental silicates coupled to anaerobic diagenesis, in particular methanogenesis, is a major source of alkalinity. Wallmann et al. (2008) reported very high rates of submarine weathering based on global methane production rates of 5 to 20 Tmol C y^{-1}, which are much higher than present-day estimates (2.8 Tmol C y^{-1}; Egger et al., 2018; 0.3-2.1 Tmol C y^{-1}; Wallmann et al., 2013). Given these uncertainties we use an estimate of 2.8 Tmol y^{-1} in our alkalinity budget of Table 3.

Anaerobic processes

Hu and Cai (2011) summarized in detail why only riverine nitrate delivery to and reduced sulfur in the ocean should be included in the alkalinity budgets for the entire ocean. The riverine nitrate delivery is well constrained at about 21 Tg Ny^{-1}, corresponding to an alkalinity production of 1.5 Tmol y^{-1}. Berner (1982) reported a sulfur burial estimate of 1.2 Tmol S y^{-1}, which relates to a net alkalinity production of 2.4 Tmol y^{-1}. Burdige (2007) revisited organic carbon burial in the ocean to 309 Tg C y^{-1}, which combined with Berners' C:S ratio of 2.8 corresponds to a reduced sulfur burial of 3.4 Tmol y^{-1} and thus alkalinity source of about 6.9 Tmol y^{-1}. For table 3 we have adopted the average, i.e. the overall alkalinity production due to the reduced sulfur burial is 4.7 Tmol y^{-1}.

Organic matter burial in marine sediments

Organic matter production generates alkalinity because of the assimilation of anions such as nitrate, phosphate and sulfate. Most of the organic matter produced in the sunlit layer is recycled, but a small fraction is ultimately buried in marine sediments. On the basis of Burdige's (2007) burial estimate of 309 Tg C y^{-1} (25.75 Tmol C y^{-1}) and Redfield organic matter ($C_{106}H_{177}O_{37}N_{16}PS_{0.4}$; Hedges et al., 2002), we arrive at a net alkalinity production of about 4.3 Tmol y^{-1}, because of nitrate (3.9 Tmol y^{-1}), phosphate (0.24 Tmol y^{-1}) and sulfate (0.2 Tmol y^{-1}) incorporation in organic matter and subsequent burial. Using a more conservative global carbon burial rate [Berner, 1982] of 126 Tg C y^{-1}, the alkalinity production would be about 1.7 Tmol y^{-1}. The average of these two estimates (3 Tmol y^{-1}) is presented in Table 3 and Figure 6B.

Riverine particulate inorganic carbon input
See text.

3.2 Alkalinity outputs

Reversed weathering
Isson and Planavsky (2018) discussed reversed weathering in detail and derived an estimate of about 1 Tmol y^{-1}, which is used here.

Carbonate burial in ocean sediments
Reported rate global carbonate burial in the open ocean vary between 11 (Milliman, 1993; Milliman and Droxler, 1996, Iglesias-Rodrigues et al., 2002; Smith, 2013; Smith and Mackenzie, 2015) and 12 Tmol C y^{-1} (Li et al., 1969; Morse and Mackenzie, 1990; Wollast, 1994): i.e. 22 to 24 Tmol y^{-1}.

Carbonate burial in ocean margin sediments
Carbonate burial in shelf and slope sediments shows a wide range, in particular because modern-day estimates (16-23 Tmol C y^{-1}) are higher than long-term burial rates (6 Tmol C y^{-1}; Morse and Mackenzie, 1990; 7.2 ±1.5 Tmol C y^{-1}, van der Ploeg et al., 2019). Ocean margin system burial rates vary between 16 (Smith, 2013), 18 (Iglesias-Rodrigues et al., 2002), 20 (Smith and Mackenzie, 2015), 21 (Milliman and Droxler, 1996; Wollast, 1994) to 23 Tmol C y^{-1} (Milliman, 1993). Recently, using a spatially explicit approach O'Mara and Dunne (2019) reported a rate of 13.7 Tmol C y^{-1} for the coastal ocean, which complemented with 4 Tmol C y^{-1} burial in slope sediments (Milliman, 1993) results in an estimate consistent with older literature. Using the modern-day carbonate burial in ocean margin (18 Tmol C y^{-1}) results in the consumption of about 36 Tmol y^{-1} of alkalinity; this estimate is presented in Table 3.
